# Effects of resistant dextrin on glycemic traits: a systematic review and meta-analysis of randomized controlled trials

**DOI:** 10.1186/s12937-026-01292-z

**Published:** 2026-03-05

**Authors:** Shanbin Chen, Linjing Zhang, Aizhen Zong, Zhaobo Gan, Xingjing Zhang, Fangling Du, He liu, Tongcheng Xu

**Affiliations:** 1 Institute of Food & Nutrition Science and Technology, Key Laboratory of Agro-Products Processing Technology of Shandong Province/Shandong Engineering Technology Research Center of food for Special Medical Purpose/Key Laboratory of Novel Food Resources Processing, Shandong Academy of Agricultural Sciences, Ministry of Agriculture and Rural Affairs, Jinan, China; 2 College of Food Science and Engineering, Bohai University, Jinzhou, China; 3 Shandong Bailongchuangyuan Biotechnology Co., Ltd, Dezhou, China; 4 Institute of Food & Nutrition Science and Technology, Shandong Academy of Agricultural Sciences, Jinan, China

**Keywords:** Resistant dextrin, Blood glucose insulin, HbA1c, HOMA-IR, Systematic review, Meta-analysis

## Abstract

**Background:**

Chronic hyperglycemia has affected billions of people currently and has emerged as an alarming public health concern for decades. Resistant dextrin (RD), an indigestible glucan with various applications in the food industry, has been reported to improve insulin sensitivity and help blood glucose control. However, the effect of RD on glucose metabolism was not systematically and comprehensively reviewed. The present study aimed to investigate the effect of RD on glycemic markers, including fasting blood glucose (FBG), fasting blood insulin (FBI), glycated hemoglobin (HbA1c), and homeostasis model assessment of insulin resistance (HOMA-IR).

**Methods:**

PubMed, Embase, Web of Science, Scopus, Cochrane library databases, and other additional records were systematically retrieved for randomized controlled trials (RCTs) on the effect of resistant dextrin intervention on FBG, FBI, HbA1c, HOMA-IR up to October 2024. Data were pooled using weighted mean difference (WMD) and 95% confidence intervals (95%CI), with P value ≤ 0.05 as statistical significance. Risk of bias was assessed with the Cochrane tool. Certainty of evidence was assessed with Grading of Recommendations Assessment, Development and Evaluation approach.

**Results:**

A total of 13 RCTs were eligible. RD significantly reduced FBG (WMD: -0.15 mmol/L, 95%CI: -0.30 to -0.00 mmol/L; *P* = 0.049; I^2^ = 77.5%; 13 trials; 952 participants) and HOMA-IR (WMD: -0.51; 95% CI: -0.93 to -0.09; *P* = 0.02; I^2^ = 25.2%; 7 trials;332 participants), while modestly improved HbA1c (WMD: -0.18%; 95%CI: -0.39 to 0.02%; *P* = 0.07; I^2^ = 62.0%; 10 trials; 788 participants). The effect of RD on FBI was not significant (WMD: -4.26 pmol/L; 95%CI: -13.05 to 4.53 pmol/L; *P* = 0.29; I^2^ = 65.0%; 8 trials; 625 participants). Subgroup analysis revealed that RD was more beneficial to overweight/obese or diabetic patients than the non-obese and non-diabetic. A dosage of 10 g per day was the most frequently used dose that showed efficacy.

**Conclusion:**

This study is so far the most extensive systematic review to evaluate the role of RD on markers of glycemic control. RD interventions can exert beneficial effects on FBG and HOMA-IR. Further high-quality and long-term studies are needed to strengthen its credibility.

**Trial Registration:**

This systematic review was registered on PROSPERO (international prospective register of systematic reviews) as CRD42023429881.

**Supplementary Information:**

The online version contains supplementary material available at 10.1186/s12937-026-01292-z.

## Introduction

Chronic hyperglycemia is one of the most common conditions that can lead to disease, such as type 2 diabetes mellitus (T2DM). It has emerged as a significant and alarming public health concern. By 2021, it was estimated that approximately 536.6 million adults living with diabetes, and far more people living with prediabetes [1]. Moreover, it was estimated that 26% individuals with impaired glucose tolerance and 50% individuals with impaired fasting glucose would progress to diabetes within a five-year period [1]. Chronic hyperglycemia can set the stage for a cascade of complications, some of which can be life-threatening. Typical complications include cardiovascular diseases, kidney diseases, and eye disorders. In 2021 alone, an estimated 6.7 million death could be fully or partly attributed to diabetes and its related complications, which account for 11.3% of all-cause mortality across the globe [2]. Consequently, it is projected that the global burden of diabetes will continue to escalate, the number of diabetes will reach 783.2 million worldwide by the year 2045 [1].

Dietary fiber enriched diets has been elucidated to yield notably superior outcomes in terms of enhancing insulin sensitivity and maintaining glucose homeostasis when compared to other commonly prescribed dietary regimens [3]. Resistant dextrin (RD), an indigestible glucan that mainly consists of β-1,6, β-1,2, α-1,6 and α,1–2 glycosidic bonds [4], exhibits outstanding water solubility, acid stability, thermal stability, rheological properties, and prebiotic functions, which makes it a promising candidate for various applications within the food industry. In the human gastrointestinal tract, RD mostly resists enzymatic hydrolysis [5, 6]. Approximately 15% of RD is digested in the small intestine, while 75% is fermented in colon [7], with around 10% is excreted in the feces [8, 9]. These properties contribute to RD’s potential for reducing body weight and enhancing satiety. Furthermore, RD’s low viscosity and high solubility facilitate rapid fermentation by intestinal microorganisms [10]. The fermentation products, mainly short chain fatty acids, offer benefits such as cholesterol reduction and improved blood glucose control in patients [11].

Randomized clinical trials (RCTs) have demonstrated that RD intake improves insulin resistance and helps blood glucose control [6, 12, 13]. However, conflicting evidence pointed out that RD intake had no significant effect on blood glucose and glycosylated hemoglobin, even though body weight was reduced in obese patients with type 2 diabetes (T2DM) after 20 weeks of intervention [14]. This inconsistency regarding the effect of RD on glycemic traits imminently needs to be verified. At present, a systematic review of RCTs only for T2DM individuals shows that RD intake significantly improved metabolic and inflammatory biomarkers [15]. Another systematic review with meta-analysis of RCTs also indicated beneficial effects of RD for weight control in overweight adults, which also supported the finding that RD helped in glycemic control [16]. However, the impact of RD on glucose metabolism-related indicators has not been systematically and comprehensively evaluated in hyperglycemia and other conditions, which also needed glycemic control, such as obesity.

This systematic review and meta-analysis aimed to explore the effects of RD on glucose metabolism indicators in the healthy, overweight or obese, T2DM and other people by evaluating the evidence from RCTs. Importantly, we analyzed the effects of various factors, such as health status, on fasting glucose, fasting insulin, HbA1c and HOMA-IR in various populations after intake of RD.

## Methods

This systematic review was registered on PROSPERO (international prospective register of systematic reviews) as CRD42023429881 and reported according to the PRISMA (preferred reporting items for systematic reviews and meta-analyses) guidelines [17].

### Search strategy

A systematic literature search was conducted in PubMed, Embase, Cochrane Library, Web of Science, Scopus up to October 2024. International Clinical Trials registry platform, European Union Clinical Trials Register and ClinicalTrials.gov were also retrieved. In addition, we conducted Google searches for gray literature. The following keywords were applied: (indigestible dextrin OR resistant dextrin OR resistant maltodextrin OR nutriose OR Fibersol-2) AND (glucose OR plasma glucose OR serum glucose OR glycemic OR glycemia OR diabetes OR diabetic OR fasting glucose OR blood glucose OR blood sugar OR insulin OR iletin OR Ins OR glycated hemoglobin OR glycosylated hemoglobin OR GHb OR HbAlc). Search strategies was described in supplementary Table 1. The references of the found papers were reviewed to find other potential studies.

### Inclusion and exclusion criteria

Study screening and selection was independently carried out by two investigators (L.Z. and S.C.). Studies met the following criteria were included: (1) Publication in English; (2) Randomized controlled with parallel or crossover design since RCTs are more likely to provide unbiased information and suitable for investigating the effect of RD on glycemic traits; (3) Participants aged between 18 and 75 years; (4) Adequate information is provided to extract or calculate the mean and standard deviations (SD) at baseline or endpoint or both; (5) Outcomes included fasting blood glucose, fasting insulin or HbA1c or HOMA-IR. The exclusion criteria were as follows: (1) Not RCT; (2) Non-original research (reviews, editorials or commentaries), abstracts only, unpublished studies, and duplicated studies.

### Data extraction and processing

Two investigators (L.Z. and S.C.) independently conducted the data extraction, and the third investigator (T.X.) made the final decision if any discrepancy. The following data were extracted: first author’s surname, year of publication, number of participants, sex, age and body mass index (BMI) of participants, regions/countries, intervention method and dose, type of placebo, study design (parallel or crossover), pathologic classification, duration of washout, the baseline and endpoint measurements of glycemic markers (FBG, FBI, HbA1c, HOMA-IR) with corresponding standard deviation (SD).

To perform the comparison, the glucose level was converted from mg/dL to mmol/L, according to the conversion formula (1 mmol/L = 18 mg/dL) and the insulin level was converted from pg/mL or µIU/mL to pmol/L, according to the conversion formulae (1 µIU/mL = 6.00 pmol/L; 1 pmol/L = 5.808 pg/mL) [18]. Changes from baseline to endpoint were used to analyze fasting blood glucose, fasting insulin, HbA1c, and HOMA-IR. When the SD was not reported, it was derived from the available data (95% confidence interval (CI), p-values, or SE) using the method suggested by the Cochrane Handbook for Systematic Review of Interventions [19]. As change from baseline was used in some of the study included, we imputed the values according to the Cochrane Handbook [19]. Means of the change and the SD for changes from baseline was imputed using a pooled correlation coefficient (R) at 0.5 as follow:

$$\:MEAN_\mathrm{change}=MEAN_\mathrm{intervention}-MEAN_\mathrm{baseline}$$;

$$\:\mathrm{S}\mathrm{D}_\mathrm{change}=\sqrt{S{D}_{baseline}^{2}+S{D}_{intervention}^{2}-\left(2\times\:R\times\:S{D}_{baseline}\times\:S{D}_{intervention}\right)}$$.

In circumstances that a control group was included in more than one comparison, SEs were inflated to avoid double counting of participants [20].

### Quality evaluation

The risk of bias was assessed using the Cochrane risk-of-bias tool for randomized trials (ROB2, Version of 22 August 2019) [21]. ROB2 employs a serial of signaling questions aimed at eliciting information relevant to five bias domains: randomization process, deviations from intended interventions, missing outcome data, measurement of the outcome, selection of the reported result. Answers to these questions were based on factual information from each study and a degree of judgement to each study. According to the responses, the risk of bias of studies in each domain was assigned into “low risk of bias”, “some concerns” or “high risk of bias”. The overall risk of bias for each study was determined by synthesizing domain-level risk of bias according to Cochrane guidelines [21]. Studies were classified as having low risk of overall bias only when all five domains were judged ‘low risk’. An overall risk of bias some concerns was assigned if one or more domains raised ‘some concerns’ while no domain was ‘high risk’. Studies received a high overall risk of bias rating if at least one domain was judged ‘high risk’, or if multiple domains with ‘some concerns’ collectively substantially undermined confidence in the results. Two researchers assessed the risk of bias independently, and any disagreement was settled by a third researcher.

### Evidence evaluation

Certainty of evidence was assessed with the online approach by Grading of Recommendations Assessment, Development and Evaluation (GRADE) on domains: study design, risk of bias, inconsistency, indirectness, imprecision, and other considerations [22]. The quality of the body of evidence was evaluated as either high, moderate, low, or very low.

### Statistical analysis

All data were processed using Stata version 16 (StataCorp LP, TX, USA). The inter-investigator reliability for literature screening and selection was checked with Cohen’s kappa test. Changes of indicators from baseline were used to estimate the effect of RD intervention on blood glucose-related indicators. Random effects model developed by DerSimonian and Laird was used to pool the overall weighted mean difference (WMD) [19]. Knapp-Hartung adjustment was applied to the standard error of the overall effect size [23]. Tau^2^, *I*^*2*^ statistics and Cochrane Q test were used to estimate the heterogeneity between the included studies, with *I*^*2*^ greater than 25% or *P* less than 0.1 representing considerable heterogeneity [24]. In addition, Funnel plot and Egger’s regression were used to exam symmetry. *P* value less than 0.05 was considered a significant publication bias, no bias was defined as a *P* value > 0.1. Sensitivity analysis was used to assess the robustness. Subgroup analysis was performed to examine potential residual confounding factors according to the intervention duration, intervention dose, inclusion age, BMI, gender, health status, and region. A dosage of 10 g RD per day was set to divide the studies into two subgroups, according to the deficient amount from dietary fiber recommendation [25]. The studies were also sub-grouped by a duration of 8 weeks, which was the most commonly applied in RCTs of dietary fiber intervention. As five of thirteen studies were from Iran, subgroup of region was divided as Iran and other country. In the study of Cai et al., the author only described the total intake amount of the mixed milk powder, and the dose of RD or inulin was not provided [6]. Therefore, the dose subgroup analysis did not include this study. When the average BMI or age were reported separately among groups or between sexes in some studies, the pooled mean BMI or age was calculated and used in corresponding analysis.

## Results

### Literature search and selection

**Fig. 1:**
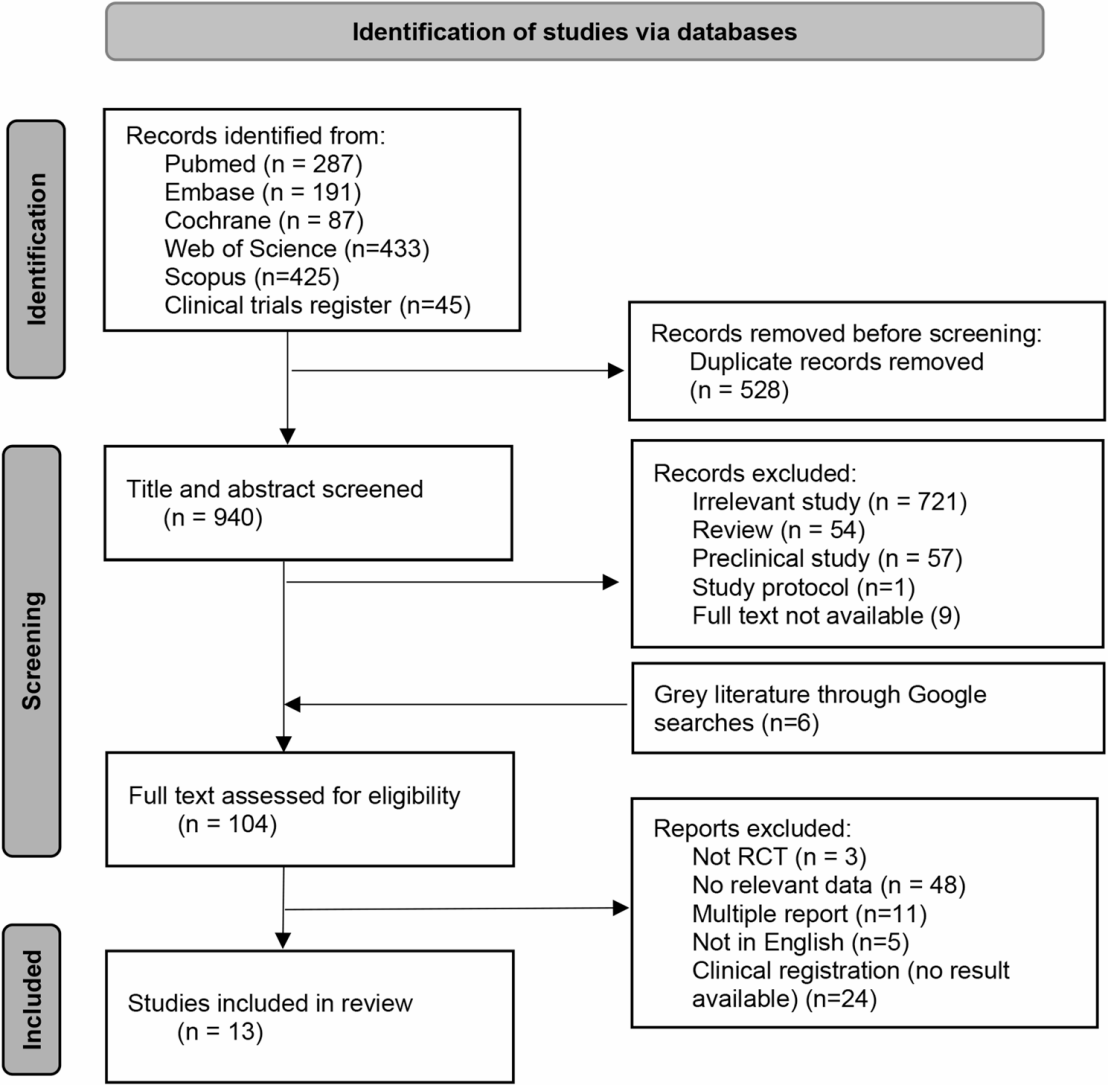
Flow diagram of the study selection procedure showing the number of eligible articles included in the meta-analysis

The systematic process for the literature searches and selection was shown in Fig. 1. Two investigators independently conducted the literature screening and selection, the results were showed in Supplementary Table 2. The inter-investigator reliability was good with Kappa statistics of 0.82. One thousand four hundred and eighty-nine articles were retrieved from databases and six grey literatures were found through Google search. Of which, 555 articles were removed after deduplication, 721 articles were not relevant to the topic, and 54 articles were reviews. After filtering out 57 preclinical studies, one protocol, and 9 studies with full text not available, 104 articles were assessed for eligibility. Studies of non-RCT, non-English published were not included. Multiple reports, studies without interested data and clinical registration with no result were excluded. The excluded studies was listed in Supplementary Table 3. Finally, 13 eligible articles with 952 participants were included in this meta-analysis.

### Study characteristics

The characteristics of the included studies are listed in Table 1. All the included study met the PICOS (population, intervention, comparison, outcome, study design) criteria. Among the 13 qualified studies, four studies from Iran included only women, and the others included both genders. The average age of the participants ranged from 27 to 61 years. The BMI ranged from 22.4 to 35.3 kg/m^2^. Study participants were overweight, obese, metabolic syndrome, T2DM, polycystic ovary syndrome, non-alcoholic fatty liver disease or health. All the 13 qualified articles were randomized controlled trials, of which 2 were cross-over trials. The intervention duration ranged from 28 to 140 days. In eight studies [6, 14, 26–31], RD was added to drinks or dairy powder; in the other studies, participants supplemented RD directly. In addition, all studies asked participants to maintain a regular diet. Thirteen studies reported fasting blood glucose, 8 reported fasting insulin, 10 reported glycated hemoglobin, and 7 reported HOMA-IR. The included studies were published from 2010 to 2022.

**Table 1:** Characteristics of the included studies

Reference	year	Location	Study design	Population		Study participants	Mean age (year)	BMI	Gender	Dose(g/d)	Intake frequency	Duration	Intervention	
				Treatment group	Control group			(kg/m^2^)			(times/day)	(day)	Treatment group	Control group
Li S	2010	China	R, DB, P	60	60	Overweight	31.0 ± 4.2	24.5 ± 0.3	F & M	34	2	84	RD	Maltodextrin
Hashizume C	2012	Japan	R, DB, P	15	15	MetS	60.7 ± 10.2	27.5 ± 2.7	F & M	27	3	84	unsweetened blended tea drink containing 9 g RD	unsweetened blended tea drink without RD
Aliasgharzadeh A	2015	Iran	R, DB, P	30	25	T2DM	49.4 ± 9.0	31.3 ± 4.8	F	10	1	56	RD	Maltodextrin
Shamasbi SG	2018	Iran	R, TB, P	31	31	PCOS	27.0 ± 6.3	26.0 ± 5.10	F	20	1	70	RD	Maltodextrin
Cai X	2018	China	R, DB, P	49	50	T2DM	60.5 ± 5.6	27.8 ± 3.6	F & M	ND	2	90	45 g milk powder with inulin and RD	45 g milk powder without inulin and RD
Farhangi MA	2019	Iran	R, TB, P	32	33	T2DM	49.0 ± 7.9	31.8 ± 4.2	F	10	2	56	RD	Maltodextrin
Mateo-Gallego R	2019	Spain	R, C	14	14	Obesity with T2DM	56.1 ± 6.3	32.1 ± 3.7	F & M	5.28	1	140	alcohol-free beer containing RD and isomaltose (16.5 g)	Alcohol-free beer
Hobden MR	2021	UK	R, C	36	36	Overweight	33.4 ± 9.3	24.9 ± 2.4	F & M	14	2	28	200 mL orange juice containing 7 g RD	200 mL Isocaloric orange juice containing 3.5 g maltodextrin
Saleh-Ghadimi S	2022	Iran	R, DB, P	33	30	Obesity with T2DM	47.0 ± 6.8	35.3 ± 6.5	F	10	1	56	RD	Maltodextrin
Kavyani M	2021	Iran	R, TB, P	18	18	NAFLD	42.95 ± 6.06	34.57 ± 2.83	F & M	10	2	84	Co-supplementing camelina oil (~ 20 g) and resistant dextrin	Co-supplementing camelina oil (~ 20 g) and maltodextrin
Chang BJ	2011	Korea	R, DB, P	48	53	Health	36.79 ± 9.40	22.39 ± 3.05	F & M	10.8	2	56	150mL yogurt with 5.4 g RD	150mL yogurt without RD
Hess AL	2020	Denmark	R, DB, P	44	42	Obesity	48.58 ± 8.76	33.77 ± 4.18	F & M	10	2	84	200 mL of semi-skimmed milk containing 5 g inulin-type fructans and 5 g RD	200 mL of semi-skimmed milk containing isocaloric maltodextrin
Kitagawa M	2020	Japan	R, DB, P	66	69	Overweight	47.1 ± 8.9	26.5 ± 1.9	F & M	15	3	84	350 mL tea beverage with 5 g RD	350 mL tea beverage without RD

*R* Randomized, *DB* Double-blind, *TB* Three-blind, *P* Parallel, *C* Crossover, *T2DM* Type 2 diabetes mellitus, *MetS* Metabolic syndrome, *PCOS* Polycystic ovary syndrome, *NAFLD* Non-alcoholic fatty liver disease, *BMI* Body mass index, *F*, Female, *M* Male, *RD* Resistant dextrin, *ND* Not described

### Risk of bias

Table 2 shows a summary of the risk of bias, as determined using the Cochrane risk-of-bias tool. Eleven studies were low risk in bias from randomization process, deviations from intended interventions, missing outcome data, measurement of the outcome, selection of the reported result. One study has imbalanced dropouts because of discontinued intervention, and the data from participants drop out was not analyzed [12]. Considering that discontinued interventions occurred in the placebo group possibly because of unsatisfactory efficacy, which would not exaggerate the effect of RD, the overall risk of bias were rated as some concern. One study did not report the detail of allocation and follow-up, which raise concern about the quality [30].

**Table 2:** Quality assessment of the included studies

Reference	Years	Domain 1. Randomization process	Domain 2. Deviations from intended interventions	Domain 3. Missing outcome data	Domain 4. Measurement of the outcome	Domain 5. Selection of the reported result	Domain 6. Overall Bias
Li S	2010	L	L	L	L	L	L
Hashizume C	2012	L	L	L	L	L	L
Aliasgharzadeh A	2015	L	SC	SC	L	L	SC
Shamasbi SG	2018	L	L	L	L	L	L
Cai X	2018	L	L	L	L	L	L
Farhangi MA	2019	L	L	L	L	L	L
Mateo-Gallego R	2019	L	L	L	L	L	L
Hobden MR	2021	L	L	L	L	L	L
Saleh-Ghadimi S	2022	L	L	L	L	L	L
Kavyani M	2021	L	SC	SC	L	SC	SC
Chang BJ	2011	L	L	L	L	L	L
Hess AL	2020	L	L	L	L	L	L
Kitagawa M	2020	L	L	L	L	L	L

The Cochrane Collaboration risk of bias tool (version 2) assessed the quality of each study. L means a low risk of bias, SC means that bias is under some concern, H means a high risk of bias

### Intervention effects

#### Fasting glucose

**Fig. 2:**
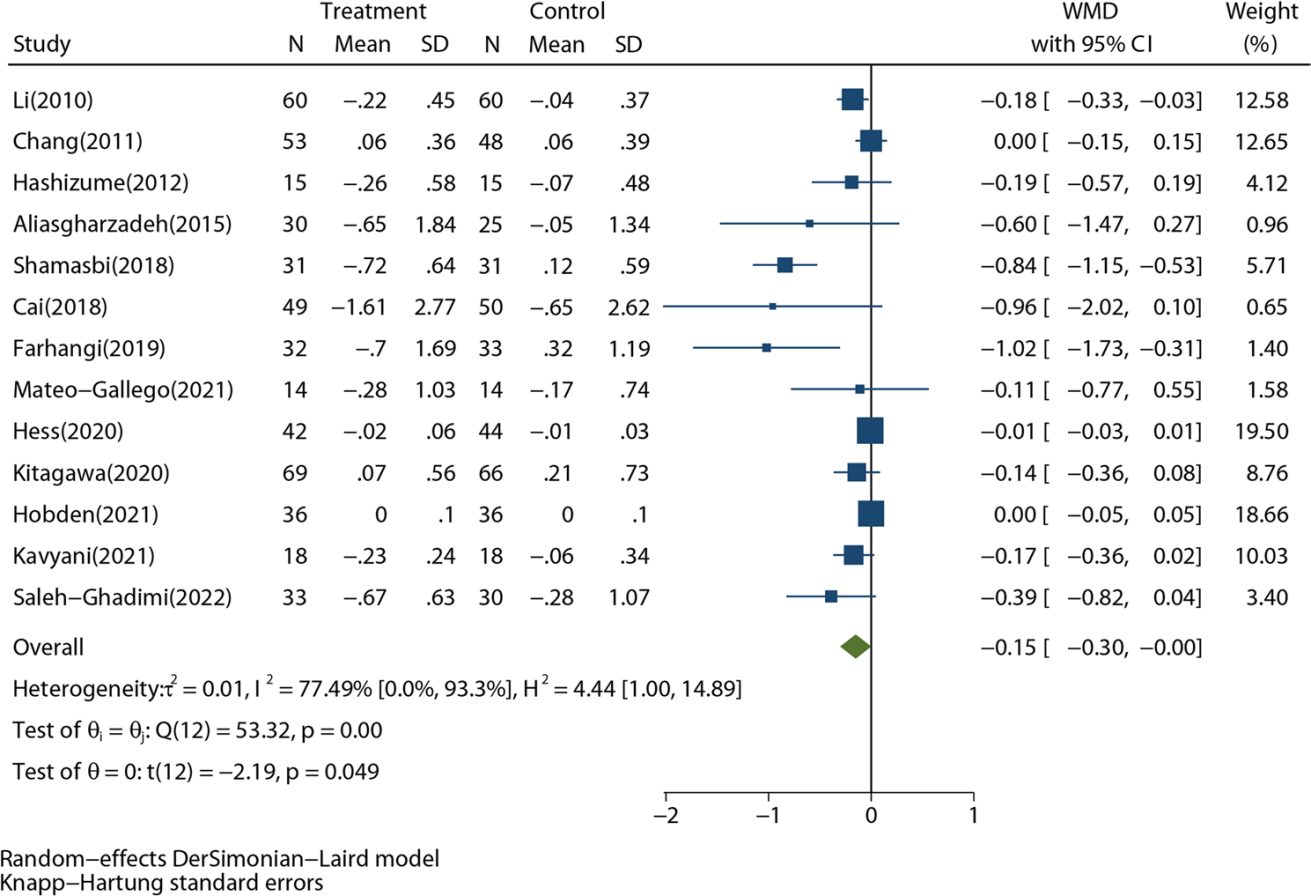
Forest plot shows the pooled effect of resistant dextrin treatment on fasting blood glucose from 13 randomized controlled trials (weighted mean difference (WMD): -0.15 mmol/L; 95% confidence interval (CI): -0.30 to -0.00 mmol/L; *P* = 0.049). Overall estimate was obtained from random-effects meta-analysis with Knapp-Hartung adjustment and represented as diamond. Closed square and ends of the line represent the WMD and 95% CIs. The size of the square represents the weight of each study

Thirteen RCTs, including 952 participants, took fasting glucose (FBG) as an outcome. The meta-analysis showed that RD decreased FBG (Fig. 2, WMD: -0.15 mmol/L, 95%CI: -0.30 to -0.00 mmol/L; *P* = 0.049). An overall significant heterogeneity of FBG among these studies was found (*I*^*2*^ = 77.49%, *P* < 0.001). The Galbraith plot (Supplementary Fig. 1) shown that studies of Farhangi [32] and Shamasbi [33] were highly heterogeneous. As sensitivity analysis showed, excluding these two studies did not reverse the pooled effect in any direction (Supplementary Fig. 2). Sensitivity analysis also indicated that Hess’s study [Hess] substantially compromised the effect of RD. The funnel plot (Supplementary Fig. 3) was not symmetrical and Egger’s test (*P* = 0.003) also indicate the asymmetry. It can also be seen in the funnel plot that the different effects of RD on FBG between T2DM studies and non-T2DM studies mainly lead to the asymmetry.

To further investigate the influence of various factors that might affect the results, subgroup analysis was conducted (Table 3). This analysis included factors of health status, baseline FBG level, BMI, age, gender, doses, intake frequency and intervention duration and region. The stratified analysis according to health conditions showed that the FBG levels significantly declined in participants with T2DM (WMD: -0.52 mmol/L; 95%CI: -0.96 to -0.08 mmol/L; *P* = 0.031). In addition, the result of the baseline FBG subgroup was similar. RD more significantly reduced the FBG in individuals with higher BMI and older adults. Interestingly, subgroup analysis regarding gender found that females benefited more from RD (WMD: -0.71mmol/L; 95%CI: -1.13 to -0.30; *P* = 0.012), while the effect was insignificant in subgroups of both genders. When the dose of RD is no higher than 10 g/d, it reduced FBG more significantly, while a dose of higher than 10 g/d did not strengthen the effect. RD intervention once daily exhibited overall significant effectiveness in reducing FBG (WMD: -0.55 mmol/L; 95% CI: -1.05 to -0.04; *P* = 0.042), and more frequent interventions did not reinforce the effect.The pooled effect of RD intervention for more than 8 weeks on FBG was more potent than that of shorter intervention duration. Interestingly, those studies from Iran showed more significant than that from other regions.

**Table 3:** Subgroup analyses by previously defined study characteristics

Subgroup	FBG							FBI						HbA1c						HOMA-IR					
	*N*	WMD (95%CI)	*P*	I2 (%)	*P*(within subgroup)		*P*(between subgroup)	*N*	WMD (95%CI)	*P*	I2 (%)	*P*(within subgroup)	*P*(between subgroup)	*N*	WMD (95%CI)	*P*	I2 (%)	*P*(within subgroup)	*P*(between subgroup)	*N*	WMD (95%CI)	*P*	I2 (%)	*P*(within subgroup)	*P*(between subgroup)
Health status																									
Non-T2DM	8	-0.11 (-0.28, 0.06)	0.176	81.5	< 0.001			5	-1.35 (-12.24, 9.55)	0.745	55.9	0.06		5	-0.06 (-0.32, 0.19)	0.521	63.3	0.028		4	-0.4 (-0.77, -0.03)	0.041	0.0	0.558	
T2DM	5	-0.52 (-0.96, -0.08)	0.031	8.7	0.353		0.020	3	-9.06 (-41.84, 23.72)	0.357	66.4	0.048	0.368	5	-0.48 (-0.67, -0.29)	0.002	0.0	0.913	< 0.001	3	-0.86 (-2.65, 0.92)	0.173	46.8	0.153	0.281
Baseline FBG level																									
< 7mmol/L	9	-0.11 (-0.27, 0.05)	0.145	78.9	< 0.001			5	-1.35 (-12.24, 9.55)	0.745	55.9	0.06		6	-0.06 (-0.27, 0.14)	0.466	54.7	0.05		5	-0.39 (-0.66, -0.12)	0.016	0.0	0.71	
≥ 7mmol/L	4	-0.61 (-1.1, -0.11)	0.03	0.0	0.448		0.003	3	-9.06 (-41.84, 23.72)	0.357	66.4	0.048	0.368	4	-0.48 (-0.73, -0.23)	0.009	0.0	0.808	< 0.001	2	-1.22 (-7.13, 4.68)	0.231	26.2	0.244	0.079
BMI																									
< 25	3	-0.05 (-0.29, 0.19)	0.493	62.0	0.072			2	0.12 (-84.45, 84.69)	0.985	75.4	0.044		2	-0.04 (-1.37, 1.28)	0.75	83.2	0.015		1	-0.54 (-1.17, 0.09)	-	-	-	
≥ 25 and < 30	4	-0.45 (-1.1, 0.19)	0.112	80.4	0.002			3	-4.08 (-18.47, 10.3)	0.347	0.0	0.473		2	-0.14 (-2.24, 1.97)	0.557	13.0	0.284		2	-0.61 (-0.89, -0.33)	0.023	0.0	0.947	
≥ 30	6	-0.23 (-0.55, 0.09)	0.126	67.1	0.009		0.082	3	-8.39 (-52.24, 35.47)	0.497	67.6	0.046	0.758	6	-0.41 (-0.82, -0.01)	0.046	41.5	0.129	0.143	4	-0.5 (-1.69, 0.7)	0.277	60.0	0.057	0.93
Age																									
< 45	4	-0.21 (-0.78, 0.37)	0.342	90.8	< 0.001			2	0.12 (-84.45, 84.69)	0.985	75.4	0.078		2	-0.04 (-1.37, 1.28)	0.75	83.2	0.015		1	-0.54 (-1.17, 0.09)	-	-	-	
≥ 45 and ≤ 60	7	-0.19 (-0.42, 0.05)	0.448	63.6	0.011			4	-8.1 (-28.36, 12.15)	0.293	56.6	0.044		7	-0.3 (-0.64, 0.03)	0.069	39.8	0.126		4	-0.5 (-1.69, 0.7)	0.277	61.3	0.057	0.93
> 60	2	-0.41 (-4.82, 4)	0.096	44.1	0.181		0.829	2	-2.52 (-98.3, 93.27)	0.795	24.6	0.249	0.659	1	-0.19 (-0.39, 0.02)	-	-	-	0.229	2	-0.61 (-0.89, -0.33)	0.023	0.0	0.947	
Gender																									
F	4	-0.71 (-1.13, -0.3)	0.012	17.2	0.318			2	-14.62 (-116.89, 87.65)	0.32	62.6	0.098		3	-0.52 (-0.95, -0.1)	0.001	0.0	0.691		1	-1.6 (-2.55, -0.65)	-	-	-	
F and M	9	-0.04 (-0.1, 0.02)	0.191	39.2	0.106		< 0.001	6	-0.52 (-8.91, 7.88)	0.881	45.3	0.104	0.104	7	-0.09 (-0.28, 0.11)	0.319	53.8	0.044	0.001	6	-0.4 (-0.63, -0.17)	0.006	0.0	0.807	0.015
Dose																									
≤ 10 g/d	7	-0.27 (-0.58, 0.05)	0.081	67.1	0.006			4	-5.42 (-29.86, 19.01)	0.531	62.9	0.044		7	-0.38 (-0.68, -0.07)	0.024	30.8	0.193		5	-0.52 (-1.34, 0.3)	0.155	49.1	0.107	
> 10 g/d	6	-0.19 (-0.49, 0.12)	0.177	85.3	< 0.001		0.637	4	-2.6 (-14.8, 9.6)	0.546	64.8	0.036	0.742	3	-0.04 (-0.33, 0.26)	0.648	66.6	0.05	0.017	2	-0.57 (-0.94, -0.19)	0.033	0.0	0.905	0.869
Frequency																									
One	4	-0.55 (-1.05, -0.04)	0.042	43.9	0.148			2	-14.62 (-116.89, 87.65)	0.32	63.4	0.098		3	-0.49 (-1.07, 0.09)	0.067	0.0	0.699		2	-0.94 (-9.45, 7.58)	0.395	72.3	0.058	
Two	7	-0.05 (-0.18, 0.07)	0.338	67.9	0.005			4	2.53 (-9.61, 14.67)	0.555	36.7	0.192		6	-0.17 (-0.47, 0.13)	0.208	72.9	0.002		4	-0.38 (-0.75, -0.01)	0.046	0.0	0.586	
Three	2	-0.15 (-0.43, 0.12)	0.090	0.00	0.824		0.009	2	-6.4 (-37.89, 25.1)	0.235	0.0	0.575	0.062	1	-0.02 (-0.38, 0.34)	-	-	-	0.071	1	-0.6 (-1.36, 0.16)	-	-	-	0.634
Duration																									
≤ 8 weeks	5	-0.14 (-0.54, 0.25)	0.372	68.4	0.005			3	-7.11 (-43.27, 29.04)	0.486	0.0	0.456		4	-0.33 (-0.93, 0.26)	0.175	3.7	0.393		1	-1.6 (-2.55, -0.65)	-	-	-	
> 8 weeks	8	-0.24 (-0.48, 0)	0.053	82.6	0.002		0.595	5	-4.18 (-12.58, 4.23)	0.24	86.2	0.001	0.742	6	-0.15 (-0.32, 0.02)	0.077	76.2	0.006	0.35	6	-0.4 (-0.63, -0.17)	0.006	0.0	0.807	0.015
Region																									
Iran	5	-0.56(-0.99,-0.12)	0.024	75.8	0.002			1	-23.35(-38.31,-8.39)	-	-	-		4	-0.57(-1.02,-0.12)	0.027	0.0	0.495		2	-0.91(-8.59,6.77)	0.374	82.2	0.018	
Others	8	-0.03(-0.09,0.03)	0.31	33.9	0.158		0.001	7	-1.53(-8.65,5.59)	0.618	44.9	0.092	0.008	6	-0.06(-0.19,0.08)	0.335	41.3	0.13	0.001	5	-0.43(-0.84,-0.01)	0.046	0.0	0.69	0.439

P (within subgroup): Cochrane Q test for within subgroup heterogeneity examination. P (between subgroup): Cochrane Q test for between subgroup heterogeneity examination

Frequency: Number of resistant maltodextrin intakes per day. *T2DM* Type 2 diabetes mellitus. *F* Female. *F* and *M* Female and male. –: not applicable

#### Fasting insulin

**Fig. 3:**
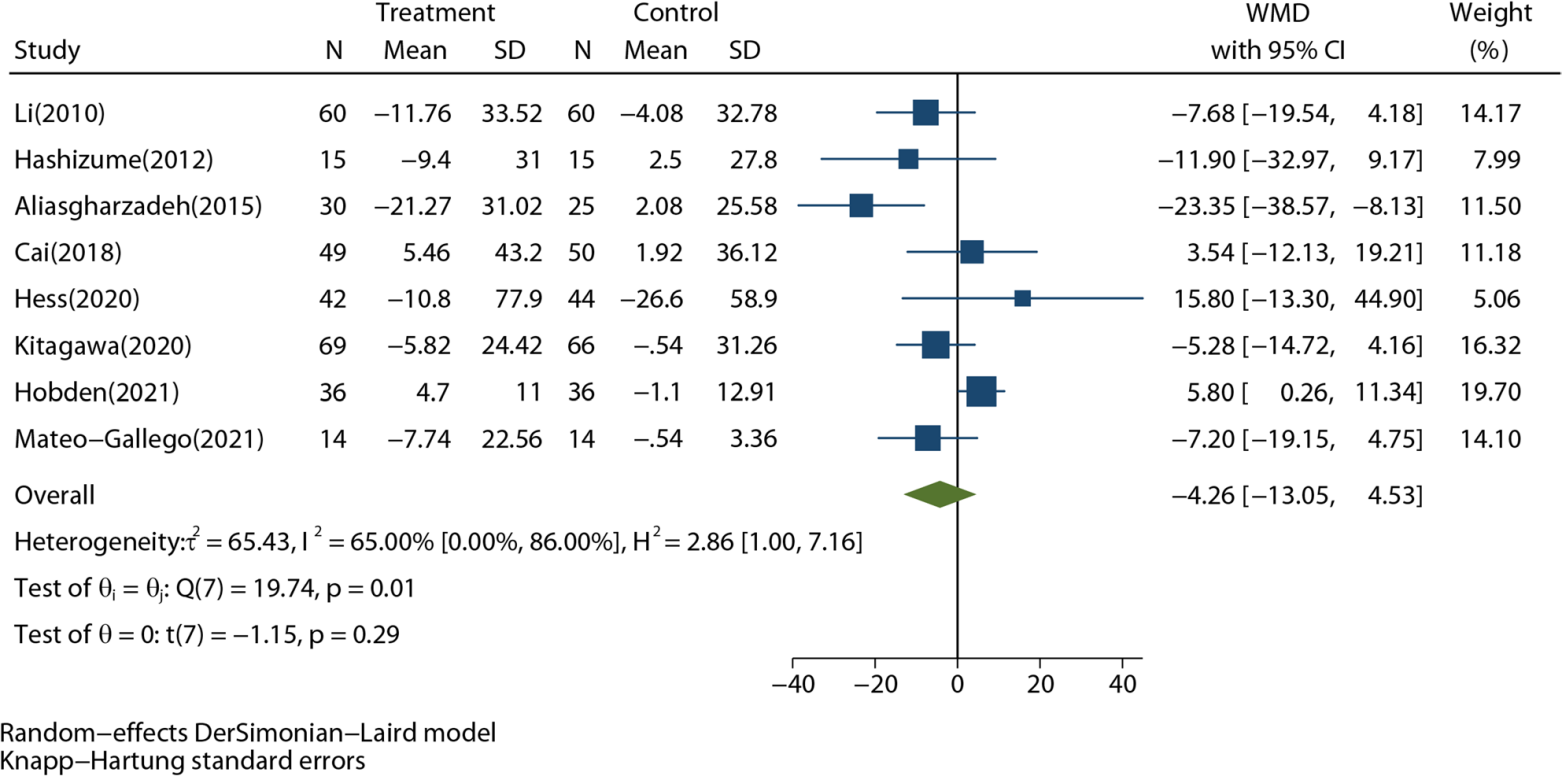
Forest plot shows the pooled effect of resistant dextrin treatment on fasting blood insulin from 8 randomized controlled trials (weighted mean difference (WMD): -4.26 pmol/L; 95% confidence interval (CI): -13.05 to 4.53 pmol/L; *P* = 0.29). Overall estimate was obtained from random-effects meta-analysis with Knapp-Hartung adjustment and represented as diamond. Closed square and ends of the line represent the WMD and 95% CIs. The size of the square represents the weight of each study

Data on fasting insulin (FBI) were reported in 8 studies involved in 625 participants. Compared with the control group, RD intervention decreased FBI, but not statistically significantly (WMD: -4.26 pmol/L; 95%CI: -13.05 to 4.53 pmol/L; *P* = 0.29) (Fig. 3). The overall heterogeneity was high (*I*^*2*^ = 65.0%, *P* = 0.01). The Galbraith plot showed that there were two studies with high heterogeneity (Supplementary Fig. 4) [12, 27]. The sensitivity analysis indicated no single study had a significant impact on the total merged effect (Supplementary Fig. 5). The funnel plot (Supplementary Fig. 6) was symmetrical, and Egger’s test did not indicate a significant publication bias (*P* = 0.221). In short, RD intervention had barely visible effect on FBI, and this result was quite stable and heterogenous.

#### Glycosylated hemoglobin (HbA1c)

**Fig. 4:**
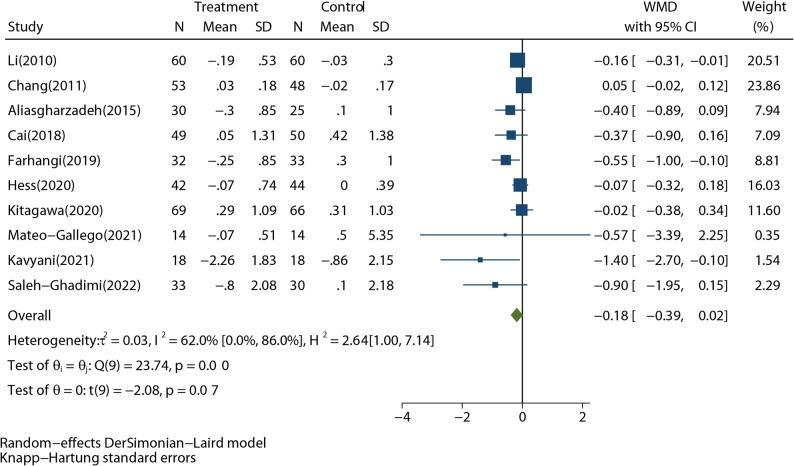
Forest plot shows the pooled effect of resistant dextrin treatment on glycosylated hemoglobin from 10 randomized controlled trials (weighted mean difference (WMD): -0.18%; 95% confidence interval (CI): -0.39 to 0.02%; *P* = 0.07). Overall estimate was obtained from random-effects meta-analysis with Knapp-Hartung adjustment and represented as diamond. Closed square and ends of the line represent the WMD and 95% CIs. The size of the square represents the weight of each study

Ten studies with 788 participants reported HbA1c outcomes. RD intake did not significantly lower the HbA1c level (WMD: -0.18%; 95%CI: -0.39% to 0.02%; *P* = 0.07) (Fig. 4). The results were heterogeneous (*I*^*2*^ = 62.0%, *P* < 0.01), and the Galbraith plot verified Chang’s study deviated from the majority (Supplementary Fig. 7). No single study had a significant impact on the pooled effect (Supplementary Fig. 8). The funnel plot was not symmetric (Supplementary Fig. 9, *P*_Egger’s test_ =0.002). As the funnel plot showed, the asymmetry was mainly attributed to the difference effect of RD between T2DM and non-T2DM.

Subgroup analysis indicated that RD intervention was more effective in T2DM or individuals with FBG≥7mmol/L, when compared to non-T2DM or individuals with euglycemia. And it was more effective in individuals with higher BMI, especially in individuals with BMI ≥ 30 kg/m^2^ (WMD: -0.41%; 95%CI: -0.82% to -0.01%; *P* = 0.046). Subgroup analysis also showed that RD significantly reduced the level of HbA1c in women (WMD: -0.52%; 95%CI: -0.95% to -0.10%; *P* = 0.001). A dose of less than 10 g/d RD was effective in reducing HbA1c level (WMD: -0.38%; 95%CI: -0.68% to -0.07%; *P* = 0.024). Studies from Iran showed more effect of RD (WMD: -0.57%; 95%CI: -1.02% to -0.12%; *P* = 0.027) than those from other countries (WMD: -0.06%; 95%CI: -0.19% to 0.08%; *P* = 0.335) in reducing HbA1c level.

#### Homeostasis model assessment of insulin resistance (HOMA-IR)

**Fig. 5:**
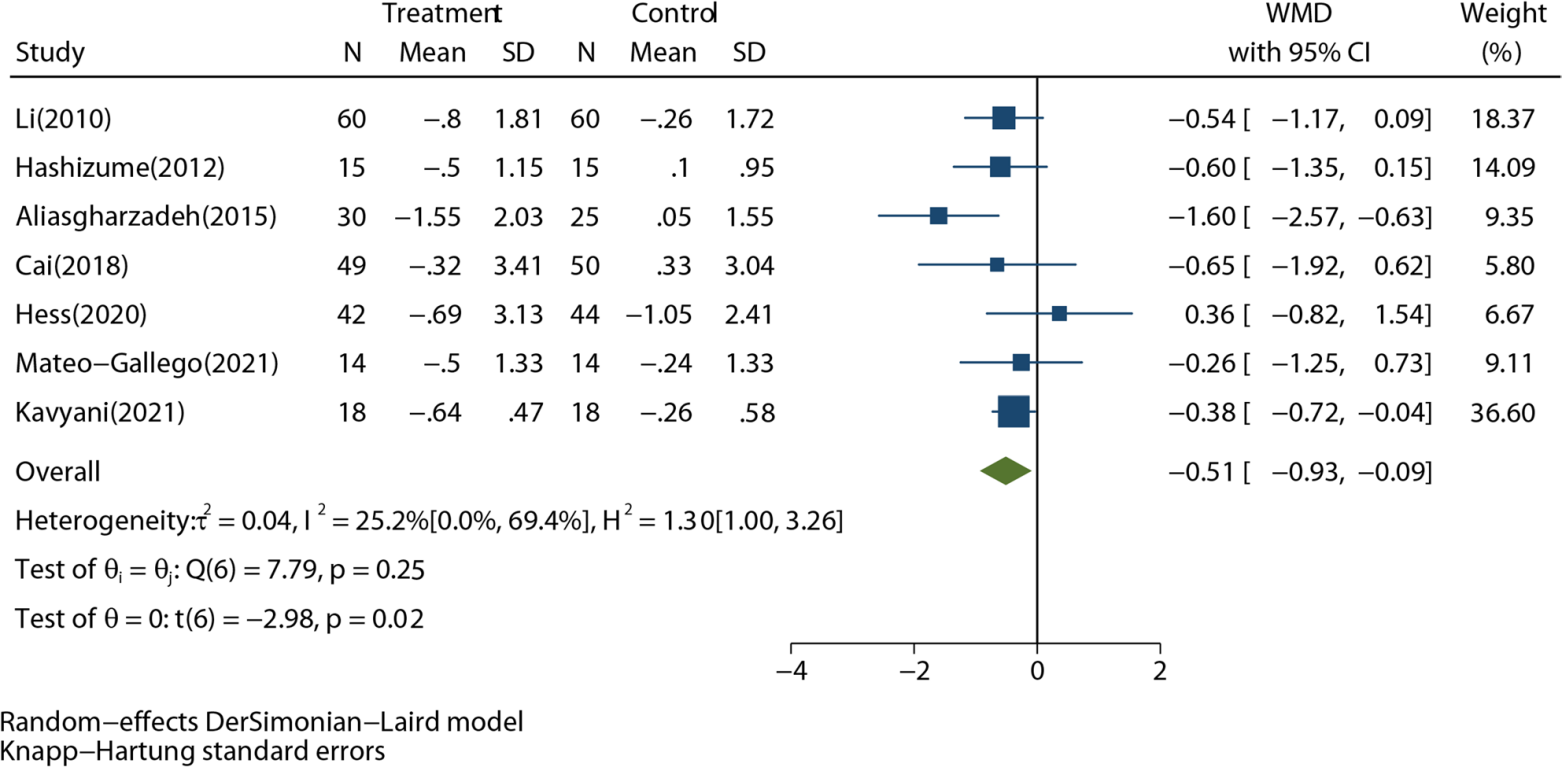
Forest plot shows the pooled effect of resistant dextrin treatment on homeostasis model assessment of insulin resistance from 7 randomized controlled trials (weighted mean difference (WMD): -0.51; 95% confidence interval (CI): -0.93 to -0.09; *P* = 0.02). Overall estimate was obtained from random-effects meta-analysis with Knapp-Hartung adjustment and represented as diamond. Closed square and ends of the line represent the WMD and 95% CIs. The size of the square represents the weight of each study

A total of seven studies with 454 participants, reported HOMA-IR outcomes. The forest plot (Fig. 5) showed the significant beneficial effect of RD intervention on HOMA-IR (WMD: -0.51; 95% CI: -0.93 to -0.09; *P* = 0.02), with no significant heterogeneity detected (*I*^*2*^ = 25.2%; *P* = 0.25). The Galbraith plot also showed no study with considerable heterogeneity (Supplementary Fig. 10). The funnel plot showed that there was no asymmetry between the studies (Supplementary Fig. 11). Egger’s test also confirmed the symmetry (*P* = 0.689). As shown in Table 3, a statistically significant effect of RD on improving HOMA-IR was found in the participants with non-T2DM (but obese, overweight, metabolic syndrome, or non-alcoholic fatty liver disease), although the effect was large in T2DM group but not significant. Similarly, the effect of RD on HOMA-IR was significant in Iran (WMD: -0.43; 95% CI: -0.84 to -0.02; *P* = 0.046, *I*^*2*^ = 0.0%) but not in other regions (WMD: -0.91; 95% CI: -8.59 to 6.77; *P* = 0.374, *I*^*2*^ = 82.2%). The heterogeneity between T2DM subgroup or other regions subgroup may make the true value obscure.

### Quality of the evidence

As shown in Table 4, the online GRADE approach was used to assess the quality of evidence. The evidence was concluded from 13 randomized controlled trials, and no serious risk of bias was found. The outcomes of FBG and HbA1c were heterogeneous, but as mentioned above, the difference between T2DM and non-T2DM was the main source of the inconsistency. So, the item inconsistency was still graded as not serious. No concern on indirectness was observed. All the trials included a relatively small number of participants, which might substantially affect the precision, so the imprecision item was rated as serious. The funnel plots of FBG and HbA1c were not symmetric, but mainly from the difference in the health status of participants, not hints of publication bias, so we did not downgrade the certainty for possible publication bias. Generally, we concluded that the evidence regarding the effect of RD intake on FBG, HbA1c, and HOMA-IR was moderate.

**Table 4:** Quality of the evidence evaluated by GRADE

Outcome	Certainty assessment							№ of patients		Absolute Effect (95% CI)	Certainty	Importance
	№ of studies	Study design	Risk of bias	Inconsistency	Indirectness	Imprecision	Other considerations	Resistant dextrin	Placebo			
FBG	13	randomised trials	not serious	not serious^a^	not serious	serious^b^	none^c^	482	470	MD **0.15 mmol/L lower** (0.3 lower to 0 lower)	⨁⨁⨁◯ Moderate^abc^	CRITICAL
FBI	8	randomised trials	not serious	Serious^d^	not serious	serious^b^	none	315	310	MD **4.26 pmol/L lower** (13.05 lower to 4.53 higher)	⨁⨁◯◯ Low^bd^	CRITICAL
HbA1c	10	randomised trials	not serious	not serious^a^	not serious	serious^b^	none^c^	400	388	Overall MD **0.18% lower** (0.39 lower to 0.02 higher)	⨁⨁⨁◯ Moderate^abc^	CRITICAL
HOMA-IR	7	randomised trials	not serious	not serious	not serious	serious^b^	none	228	226	MD **0.51 lower** (0.93 lower to 0.09 lower)	⨁⨁⨁◯ Moderate^b^	CRITICAL

*CI* Confidence interval, *MD* Mean difference

^a^Although the I^2^ and Cochrane Q test indicate a high heterogeneity, the source of the inconsistency mainly came from the non-T2DM studies, and the effect of resistant dextrin on FBG of T2DM was quite consistent. As T2DM patients are who need glycemic control, the inconsistency item was rated as not serious

^b^The number of participants included in the trials was small, which might substantially affect the precision

^c^The funnel plot was not symmetric, but mainly from the difference in the health status of participants, not hints of publication bias

^d^The I^2^ and Cochrane Q test indicate a high heterogeneity in both non-T2DM and T2DM subgroup

## Discussion

The present study investigated the effect of RD intervention on adults, including overweight, obese, metabolic syndrome, polycystic ovary syndrome, non-alcoholic fatty liver disease, and T2DM. An overall effect from 13 RCTs with 952 participants showed that RD intake had significant improvement in FBG and HOMA-IR, a modest effect on HbA1c, but no effect on FBI. The certainty of the evidence was graded as low to moderate. There was significant heterogeneity among the trials on FBG, FBI, and HbA1c. As summarized in Table 1, the participants included in each study were quite different in health status, age, BMI, gender and the intervention were also differed in intake dose, frequency, and duration. Hence, a random-effects model with variance adjusted by the Hartung-Knapp modification was applied to achieve a robust effect size. Even so, a significant effect of RD intervention on FBG and HOMA-IR was observed. Although the overall effect of RD on HbA1c was not significant, the subgroup revealed its effectiveness in T2DM who were urgently in need of practical glycemic control measurement. Effect of RD intervention on FBI was minimal. In short, this meta-analysis demonstrates the beneficial effect of RD on markers of glycemia, particularly FBG, HbA1c, and HOMA-IR.

RD, as a dietary fiber with suitable properties for food processing, has been intensely studied since it was first prepared by Matsutani Chemical Industry Co., Ltd. in the 1980s. In the past dozen years, It’s effects on human health were also evaluated by clinical trials. Most of the RCTs focused on the effect of RD on individuals with metabolic dysfunction, such as obesity and diabetes. Colantonio and colleagues summarized the effect of prebiotics on metabolic and inflammatory biomarkers in T2DM [15]. The evidence supported the health benefits of prebiotic, of which RD showed as one of the most promising prebiotics. Mukai et al. conducted a meta-analysis of RCTs on the effect of RD for weight loss in overweight adults. Although only three trials were included, the RD’s beneficial effect for weight loss also hinted its potential on glycemic control. More recently, reviews have summed up the clinical effect of RD [11, 34]. The present systematic review and meta-analysis of RCTs provided a quantified and detailed inspection on the hypoglycemic effect of RD.

As the meta-analysis showed, RD intake was of benefit to controlling blood glucose, especially in T2DMs. The subgroup analysis revealed that RD supplementation for T2DM significantly changed FBG by -0.52 mmol/L (95%CI: -0.96, -0.08) and HbA1c by -0.48% (95%CI: -0.67, -0.29). HOMA-IR was also improved by -0.51 (95%CI: -0.93, -0.09), which might be driven by RD’s effects on glucose rather than insulin. It was obvious that RD’s effect could not compare to hypoglycemic drugs, which changed HbA1c level by -0.60% to -1.48% in drug-naive diabetes [35]. As a dietary fiber and prebiotic, RD’s hypoglycemic effect is considerable among dietary treatments, which is also a key component of diabetes management. As Jing’s meta-analysis reported, HbA1c of T2DM was reduced by a low glycemic index diet (WMD: -0.37%, 95% CI: -0.83%, 0.10%) or low carbon diet (WMD: -0.69%, 95% CI: -1.32%, -0.06%) [36]. The dietary fiber overall was reported to reduce HbA1c in diabetes (WMD: -0.2%, 95% CI: -0.33%, -0.07%) [37], while inulin, a widely accepted prebiotic, reduced HbA1c in individuals with chronic hyperglycemia (WMD: − 0.58%; 95% CI − 0.83, − 0.32%) [38]. Compared to these widely accepted dietary management for diabetes, the effect of RD on glycemic indicators is not inferior. In addition, RD is Generally Recognized as Safe by FDA and RD supplementation is cheap and widely available. Given these considerations, increased RD intake is recommended for dietary management of diabetes.

The pooled data showed that the fasting level of insulin was not significantly affected by RD intake. Considering the effects on reducing blood glucose of RD, it was reasonable to presume that RD intake will improve the glucose metabolism in a manner independent of stimulated the secretion of insulin, a potential mechanism by which dietary fiber improved glucose metabolism [39]. This view could be corroborated by inspecting the results from postprandial studies. However, the available data was not so plentiful. Although, it was reported that milk powder co-supplemented with inulin and resistant dextrin intervention for 12 weeks was effective in increasing postprandial insulin level of T2DM [6], the direct stimulation of insulin was not observed. In the clinical trial where healthy participants received a single serve of formula containing approximate 7.5 g RD (replacing 30% of the maltodextrin of the original formula), the postprandial insulin was dramatically decreased, while the postprandial plasma glucose unaltered [40]. In another RCT, where healthy or overweight participants daily consumed 14 g RD for 28 days, the RD intake before breakfast did not alter the postprandial blood insulin level, while the blood glucose level was decreased [27].

As dietary fiber, resistant dextrin resisted the host’s digestive enzymes, but could be utilized by gut microbe and acted as a regulator of gut microbiome. It was more and more recognized that the gut microbiome played an essential role in the development and management of the insulin resistance, a complex condition where body’s sensitivity to insulin reduced [41]. Insulin resistance was the primary pathophysiology underlying hyperglycemia, especially type 2 diabetes [42]. Animal studies have been showed that RD could alleviate high-fat-high-fructose diet-induced insulin resistance by promoted hepatic IRS-1-PI3K-AKT pathway, an insulin signaling pathway by which insulin facilitated hepatic utilization of glucose tong and reduces gluconeogenesis [43–45]. The alleviation on the insulin resistance of RD is closely related to its role in regulating gut microbiome. It was widely accepted that the dysbiosis of gut microbiome took an important part in the development of insulin resistance, possibly via influencing the host metabolization of major nutrients, mediating chronic inflammatory responses through intestinal flora metabolites (such as lipopolysaccharides, short-chain fatty acids, bile acids, etc.), destroying the intestinal barrier or affecting the normal function of the gut-brain axis [46] Several studies have reported the influence of RD on the gut microbiome. Mice supplemented with RD had a significantly reduced *Firmicutes/Bacteroidetes* ratio and increased metabolically beneficial bacteria such as *Prevotella* and *Akkermansia*, when compared to the high-fat-high-fructose-diet [45]. Studies of human experiments found that species of *Parabacteroides* genus, which was associated with decreased weight gain and reduced hyperglycemia, were the most distinctive flora after RD supplementation [47–49]. In addition, RD was also reported to regulate the circulation or enteric levels of intestinal flora metabolites. As a prebiotic, RD was metabolized by the gut microbes, and the products, short chain fatty acids(SCFAs, mainly acetate, propionate and butyrate), were intensively reported to inhibition inflammation [50]. RD was also reported to reduce circulation lipopolysaccharide in T2DM, a potent activator of inflammation from mostly gram-negative bacteria inhabiting in the gut [12, 32, 51]. This effect probably contributed to its alleviation of inflammation in T2DM, as indicated by decreasing circulation proinflammatory cytokines such as TNF-α, IL-1β and enhancing anti-inflammatory cytokines such as IL-10 [12, 51]. Hence, RD helped improve hyperglycemia as inflammation played a causal role in the development of insulin resistance, particularly in adipose, skeletal muscle, and liver [43]. Besides, SCFAs also served as important appetite regulatory molecules in the cross talk between gut microbiome and host. Acetate was reported to play a direct role in central appetite suppression [52]. Propionate was reported to stimulate peptide YY and glucagon like peptide-1 (GLP-1) secretion in overweight adults [53]. Animal studies showed that ingestion of RD could increase the secretion and production of GLP-1 [54]. RD supplementation was also reported to increase satiety in overweight males [55]. Consistent with these findings, meta-analysis proved that RD supplementation helped in weight loss and remission of abdominal adipose accumulation, an efficacious clinical measure to improve insulin sensitivity and glucose metabolism [16, 56, 57]. In addition, GLP-1 was reported to enhance insulin sensitivity.

Besides, as a glucan with complex branching structure, RD also possessed ability similar to acarbose (an oral antidiabetic agents) in binding to α-glucosidase, inhibiting the hydrolysis by α-glucosidase or α-amylase [58]. In vitro study showed that RD inhibited the activity of α-glucosidase and α-amylase in a dose-dependent manner [59]. Though the inhibition rate were significantly lower than the acarbose, the acceptable dose of RD was higher than acarbose, and this effect in fact helped glycemia control.

The subgroup analysis indicated that RD was more effective on the glycemic markers of T2DM, especially those with higher baseline FBG. RD was also more effective on the glycemic marker in participants with a higher BMI, an index of adipose accumulation. Adipocytes have been reported to secrete hormones such as resistin to inhibit insulin sensitivity [60]. In addition, obesity was associated with chronic low-grade systematic inflammation, which also contributed to insulin resistance. Based on the reports that RD intake helped getting rid of excess adipose and reducing inflammation [16, 56], it was reasonable to speculate that RD have a more obvious effect on controlling blood glucose in obese patients, whose blood glucose measures were higher and had more room to improve. Interestingly, studies with females reported a more significant effect than studies with mixed sexes. We noticed that the trials in females were all in Iran, and the compliance seemed not better in females as the dropout rate was almost equal. We still did not find exact explanation to this difference. The finding that RD intake significantly reduced free testosterone in women with polycystic ovarian syndrome suggested that this gender difference might be associated with sex hormones, as overexposure to testosterone was directly linked with insulin resistance and hyperinsulinemia [8, 61]. As for dosage, RD intake at a dose of 10 g per day was sufficient to observe the benefits of glycemic control, while a higher dose did not demonstrate additional benefit.

Our systematic review and meta-analysis has several advantages. Firstly, we conducted a repeatable search from multiple databases and screening process for the literature, and included all accessible relevant clinical trials of RD intake on blood glucose control. Secondly, quality of all the studies was assessed with the Cochrane risk of bias tool and the evidence was qualified with GRADE approach, that made this work more practical. On the other hand, some of our analyses have limitations. Firstly, a limited number of studies was available, some issues were not able to be addressed, such as whether the effect varied between nationalities, genders or RDs from different origin. Secondly, over a third of the included studies were from one group of Tabriz University of Medical Sciences from Iran. Though no clue of bias was noticed, a simplex source of the data indeed compromised the generalizability and credibility of the evidence.

## Conclusion

The evidence by now showed that the RD intake had a significant effect on improving FBG and HOMA-IR, especially in individuals with diabetes or higher BMI. However, the relevant rigorous clinical trials are still insufficient, especially in populations urgently needing glycemic control measures, such as the elderly and gestational diabetes. And existing studies on specific populations are insufficient to yield conclusive evidence. As enhanced population homogeneity is paramount to improve result interpretability and generalizability, we strongly recommended future clinical trials focusing on study population who urgently need glycemic control. Furthermore, an intervention regimen regarding the standardized dosage of RD is not clarified. Hence, dedicated dose-finding studies are also warranted to establish efficacy and safety thresholds specifically within hyperglycemia. The postprandial glycemic response after RD intake also needs to be fully elucidated. Generally, although this study found some benefits of RD, further high-quality and long-term studies aimed to address these issues are needed to strengthen its credibility.

## Supplementary Information

Supplementary Material 1

## Data Availability

No datasets were generated or analysed during the current study.
