# Supplementary material for: Effects of resistant dextrin on glycemic traits: a systematic review and meta-analysis of randomized controlled trials
Source: Nutr J. 2026 Mar 5;25:45. doi: 10.1186/s12937-026-01292-z (PMC13072545; doi:10.1186/s12937-026-01292-z)
Supplement: Supplementary file 1 — Supplementary Material 1 [file 12937_2026_1292_MOESM1_ESM.zip › Supplementary Table 2 Inter-investigator reliability for literature screening and selection..docx]

Supplementary Table 2 Inter-investigator reliability for literature screening and selection.

| L.Z.\S.C. | Exclude after deduplicate | Exclude after title and abstract screen | Exclude after full text assessed | Included | Total |
| --- | --- | --- | --- | --- | --- |
| Exclude after deduplicate duplicate | 488 | 23 | 27 | 0 | 538 |
| Exclude after title and abstract screen | 11 | 803 | 22 | 1 | 837 |
| Exclude after full text assessed | 29 | 33 | 45 | 0 | 107 |
| Included | 0 | 0 | 1 | 12 | 13 |
| Total | 528 | 859 | 95 | 13 | 1495 |

Cohen’s Kappa=0.82
