# Supplementary material for: Effects of resistant dextrin on glycemic traits: a systematic review and meta-analysis of randomized controlled trials
Source: Nutr J. 2026 Mar 5;25:45. doi: 10.1186/s12937-026-01292-z (PMC13072545; doi:10.1186/s12937-026-01292-z)
Supplement: Supplementary file 1 — Supplementary Material 1 [file 12937_2026_1292_MOESM1_ESM.zip › Supplementary Table 1 Search strategy.docx]

Supplementary Table 1 Search strategy.

| Database | Search Strategy |
| --- | --- |
| PUBMED | ((indigestible dextrin) OR (resistant dextrin) OR (resistant maltodextrin) OR (nutriose) OR (Fibersol-2)) AND ((glucose OR (plasma glucose) OR (serum glucose) OR glycemic OR glycemia OR diabetes OR diabetic OR (fasting glucose) OR (blood glucose) OR (blood sugar) OR insulin OR iletin OR Ins OR (glycated hemoglobin) OR (glycosylated hemoglobin) OR GHb OR HbA1c) |
| Embase | ((indigestible dextrin) OR (resistant dextrin) OR (resistant maltodextrin) OR (nutriose) OR (Fibersol-2)) AND ((glucose OR (plasma glucose) OR (serum glucose) OR glycemic OR glycemia OR diabetes OR diabetic OR (fasting glucose) OR (blood glucose) OR (blood sugar) OR insulin OR iletin OR Ins OR (glycated hemoglobin) OR (glycosylated hemoglobin) OR GHb OR HbA1c) |
| Cochrane library | #1 (indigestible dextrin):ti,ab,kw |
|  | #2 (resistant dextrin):ti,ab,kw |
|  | #3 (resistant maltodextrin):ti,ab,kw |
|  | #4 (nutriose):ti,ab,kw |
|  | #5 (Fibersol-2):ti,ab,kw |
|  | #6 (glucose):ti,ab,kw |
|  | #7 (plasma glucose):ti,ab,kw |
|  | #8 (serum glucose):ti,ab,kw |
|  | #9 (glycemic):ti,ab,kw |
|  | #10 (glycemia):ti,ab,kw |
|  | #11 (diabetes):ti,ab,kw |
|  | #12 (diabetic):ti,ab,kw |
|  | #13 (fasting glucose):ti,ab,kw |
|  | #14 (blood glucose):ti,ab,kw |
|  | #15 (blood sugar):ti,ab,kw |
|  | #16 (insulin):ti,ab,kw |
|  | #17 (iletin):ti,ab,kw |
|  | #18 (glycated hemoglobin):ti,ab,kw |
|  | #19 (glycosylated hemoglobin):ti,ab,kw |
|  | #20 (GHb):ti,ab,kw |
|  | #21 (Ins):ti,ab,kw |
|  | #22 (HbA1c):ti,ab,kw |
|  | #23 #1 OR #2 OR #3 OR #4 OR #5 |
|  | #24 #6 OR #7 OR #8 OR #9 OR #10 #11 OR #12 OR #13 OR #14 OR #15 #16 OR #17 OR #18 OR #19 OR #10 OR #21 OR #22 |
|  | #25 #23 AND #24 |
| Web of science | #1 (((TS=(Resistant *dextrin*)) OR TS=(ingestible *dextrin*)) OR TS=(nutriose)) OR TS=(Fibersol-2)  #2 (((((((((((TS=(glucose)) OR TS=(glycemi*)) OR TS=(diabetes)) OR TS=(diabetic)) OR TS=(blood sugar)) OR TS=(insulin)) OR TS=(iletin)) OR TS=(Ins)) OR TS=(glycated hemoglobin)) OR TS=(glycosylated hemoglobin)) OR TS=(GHb)) OR TS=(HbA1c)  #1 AND #2 |
| Scopus | (TITLE-ABS-KEY (resistant AND *dextrin*) OR TITLE-ABS-KEY (ingestible AND *dextrin*) OR TITLE-ABS-KEY (nutriose) OR TITLE-ABS-KEY (fibersol-2)) AND (TITLE-ABS-KEY (glucose) OR TITLE-ABS-KEY (glycemi*) OR TITLE-ABS-KEY (diabetes) OR TITLE-ABS-KEY (diabetic) OR TITLE-ABS-KEY (blood AND sugar) OR TITLE-ABS-KEY (insulin) OR TITLE-ABS-KEY (iletin) OR TITLE-ABS-KEY (glycated AND hemoglobin) OR TITLE-ABS-KEY (glycosylated AND hemoglobin) OR TITLE-ABS-KEY (ghb) OR TITLE-ABS-KEY (hba1c) OR TITLE-ABS-KEY (ins)) |
