# Supplementary figures and images for: Effects of resistant dextrin on glycemic traits: a systematic review and meta-analysis of randomized controlled trials
Source: Nutr J. 2026 Mar 5;25:45. doi: 10.1186/s12937-026-01292-z (PMC13072545; doi:10.1186/s12937-026-01292-z)

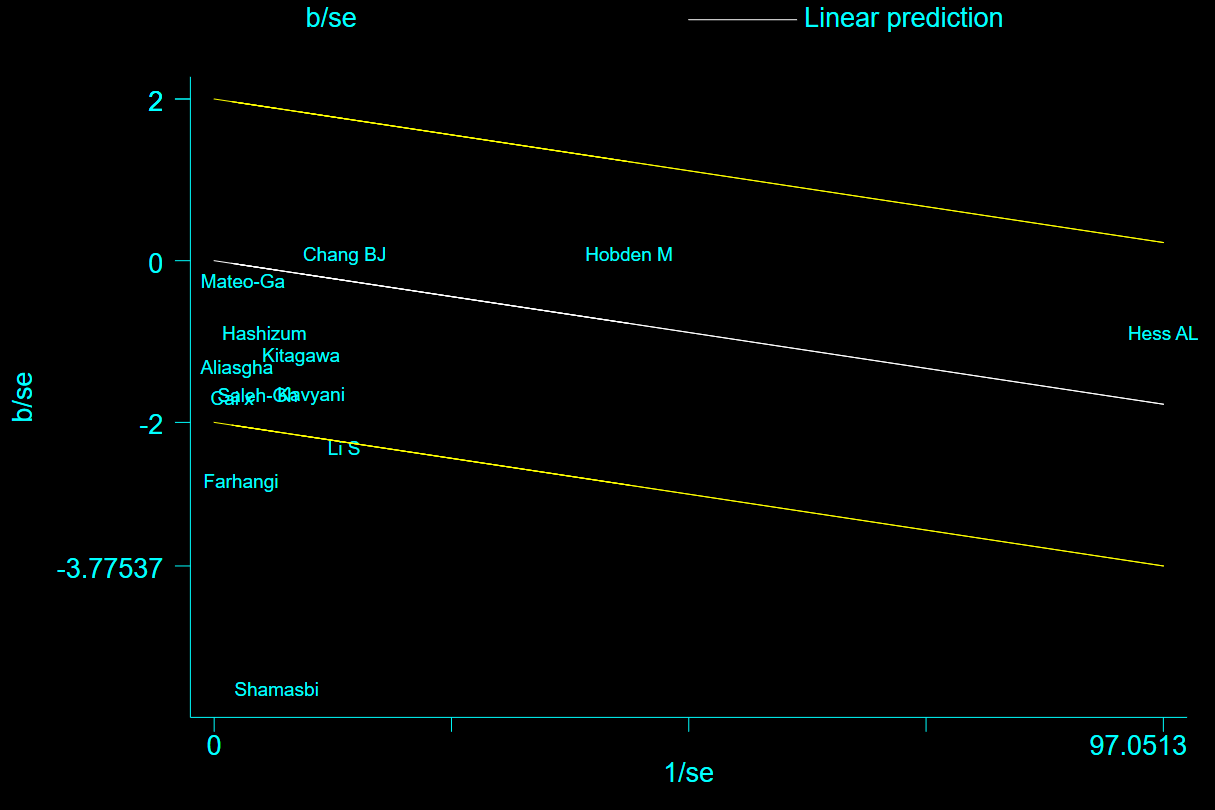

Supplement: Supplementary file 1 — Supplementary Material 1 [file 12937_2026_1292_MOESM1_ESM.zip › Supplementary Figure 1 FBG galb.tif]

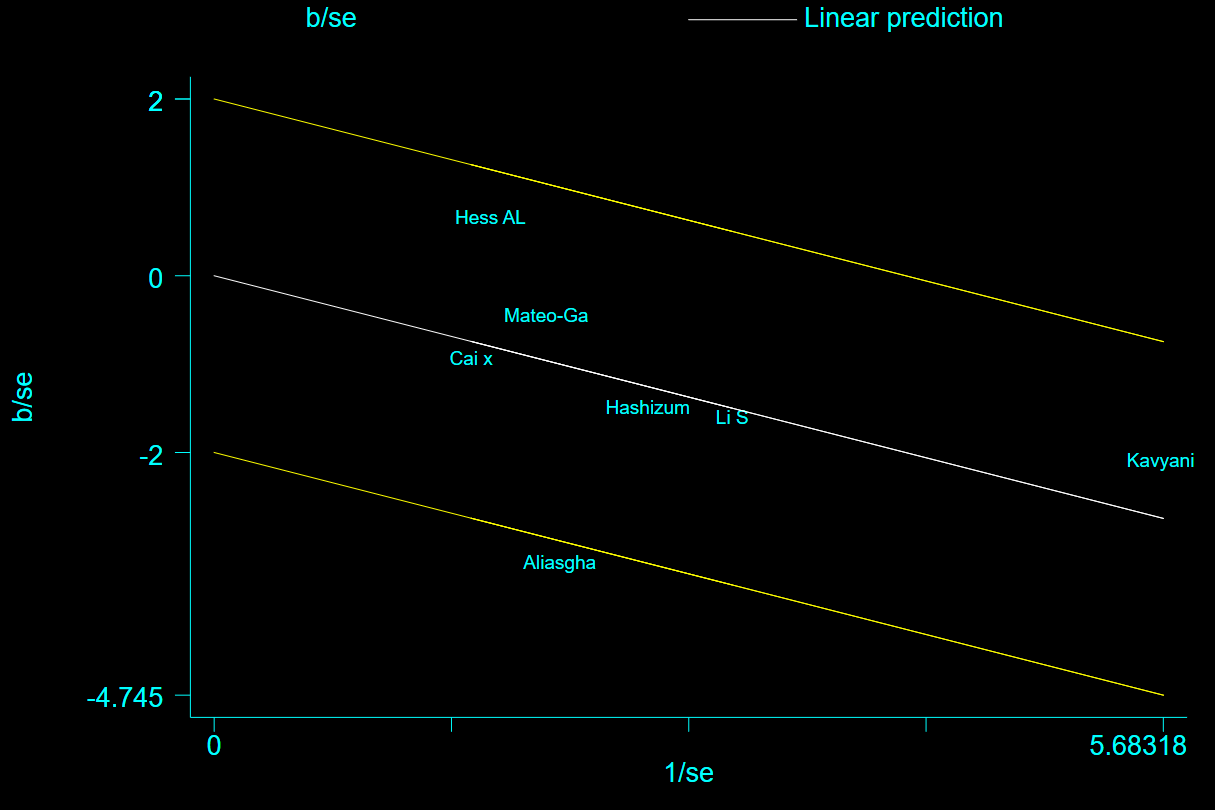

Supplement: Supplementary file 1 — Supplementary Material 1 [file 12937_2026_1292_MOESM1_ESM.zip › Supplementary Figure 10 HOMA-ir galb.tif]

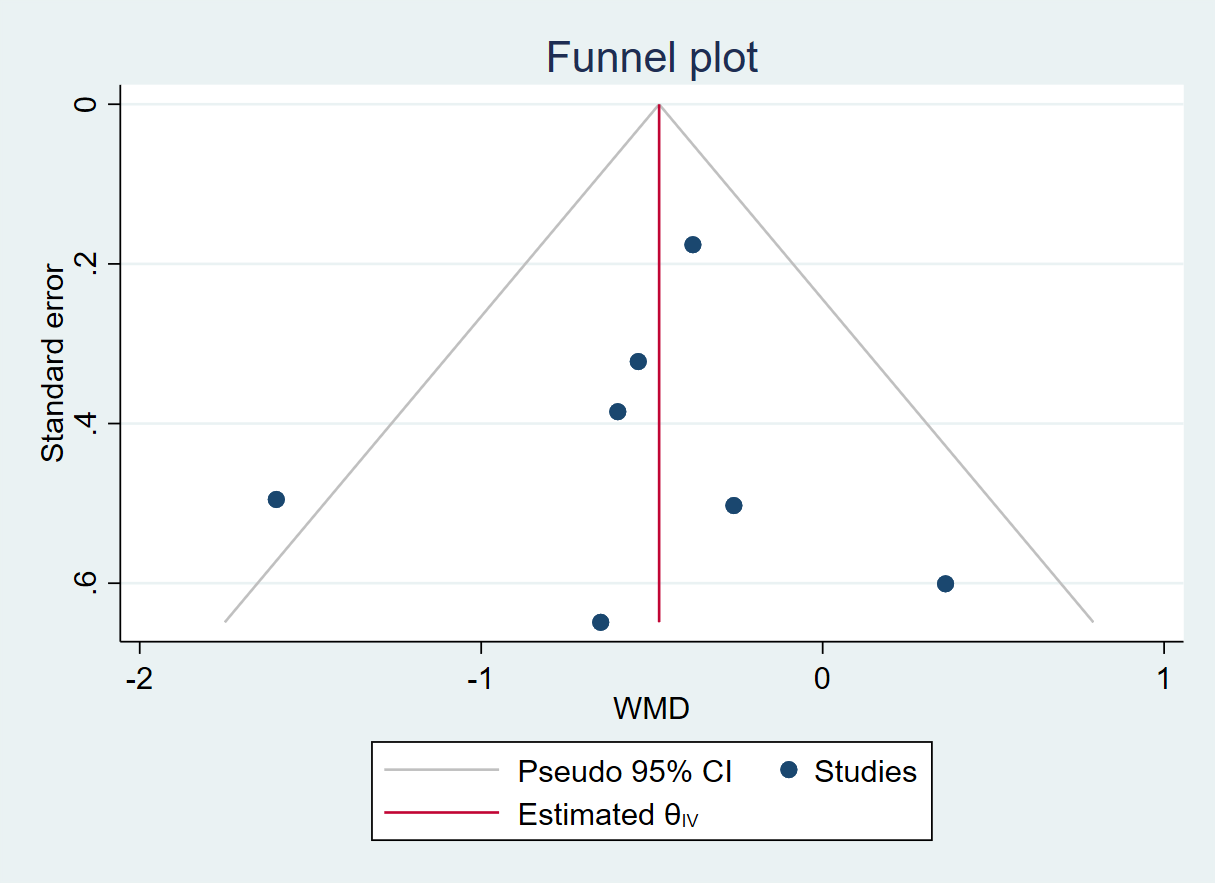

Supplement: Supplementary file 1 — Supplementary Material 1 [file 12937_2026_1292_MOESM1_ESM.zip › Supplementary Figure 11 HOMA-ir funnel.tif]

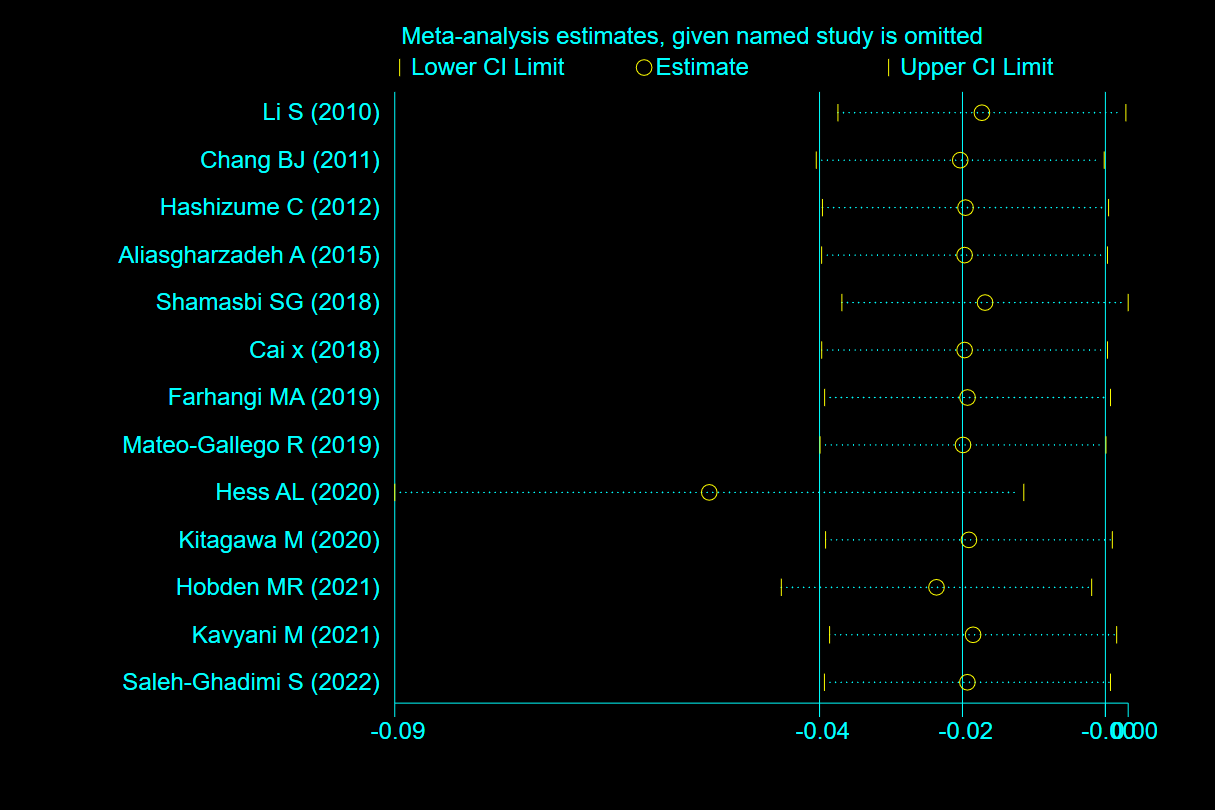

Supplement: Supplementary file 1 — Supplementary Material 1 [file 12937_2026_1292_MOESM1_ESM.zip › Supplementary Figure 2 FBG sensitive.tif]

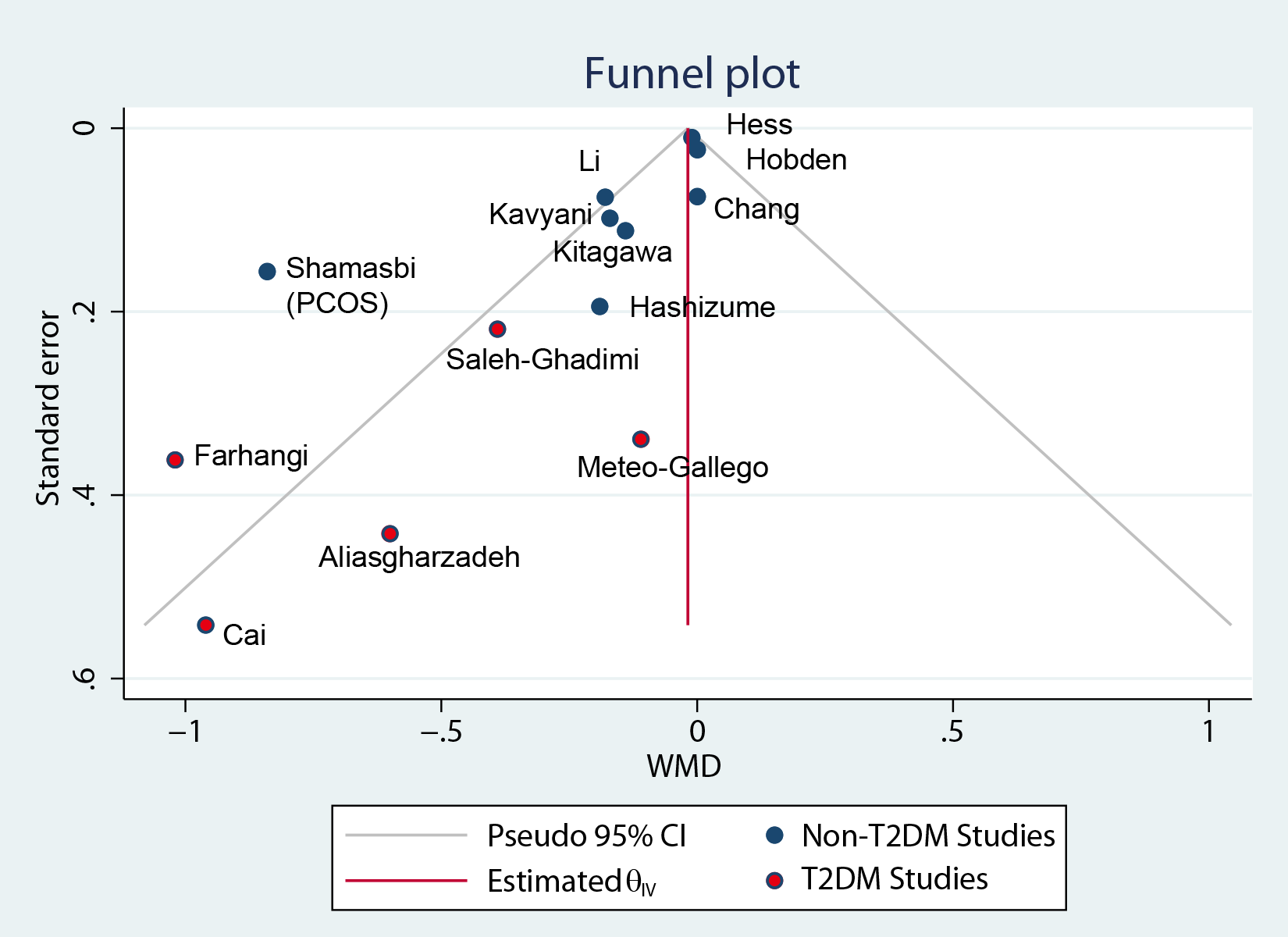

Supplement: Supplementary file 1 — Supplementary Material 1 [file 12937_2026_1292_MOESM1_ESM.zip › Supplementary Figure 3 FBG funnel.tif]

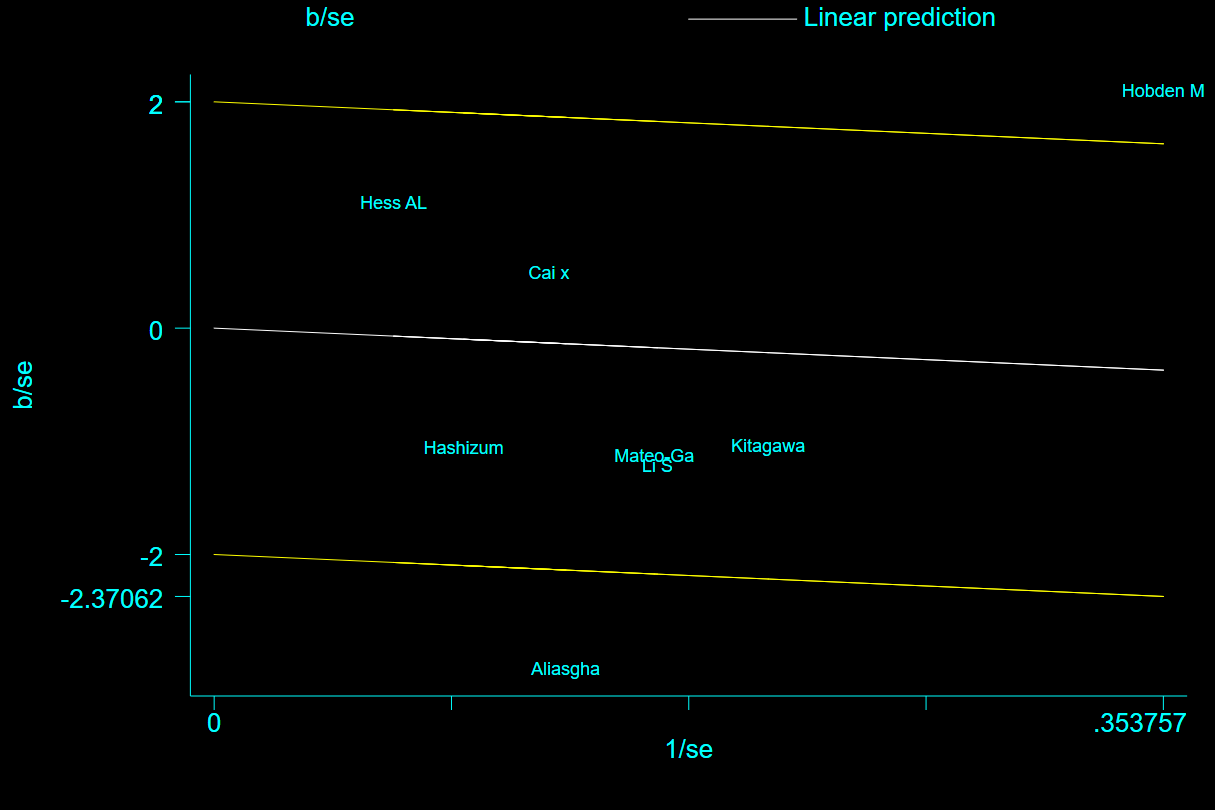

Supplement: Supplementary file 1 — Supplementary Material 1 [file 12937_2026_1292_MOESM1_ESM.zip › Supplementary Figure 4 FBI galb.tif]

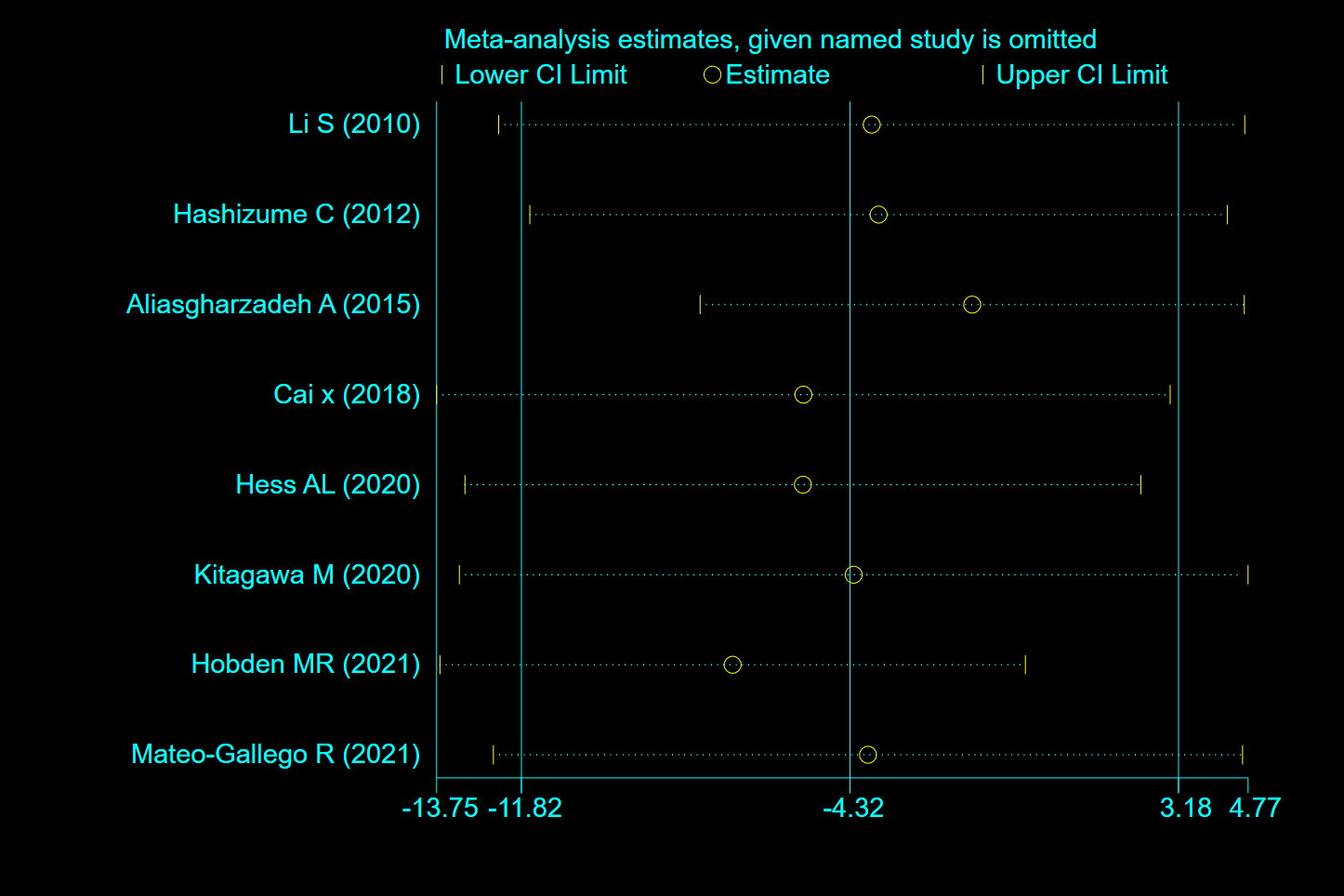

Supplement: Supplementary file 1 — Supplementary Material 1 [file 12937_2026_1292_MOESM1_ESM.zip › Supplementary Figure 5 FBI sensitive.tif]

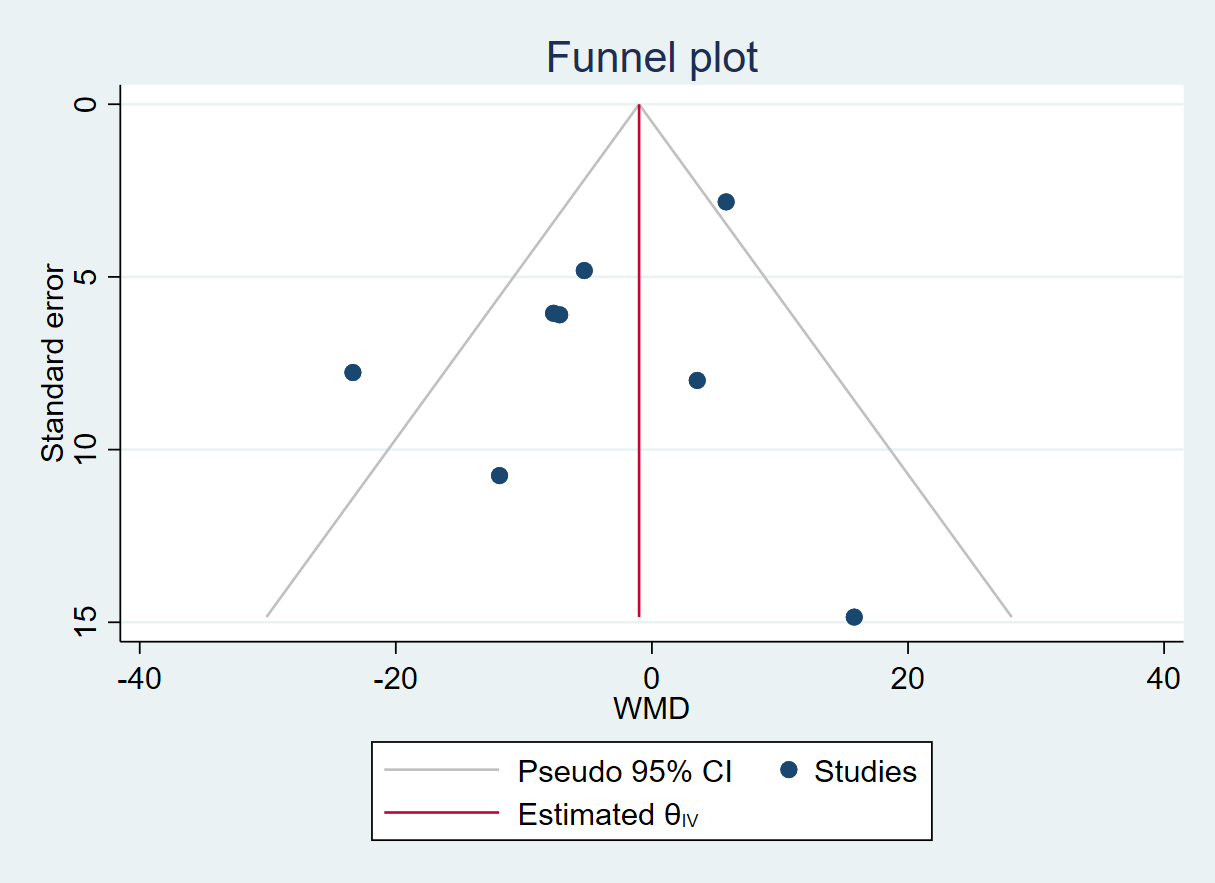

Supplement: Supplementary file 1 — Supplementary Material 1 [file 12937_2026_1292_MOESM1_ESM.zip › Supplementary Figure 6 FBI funnel.tif]

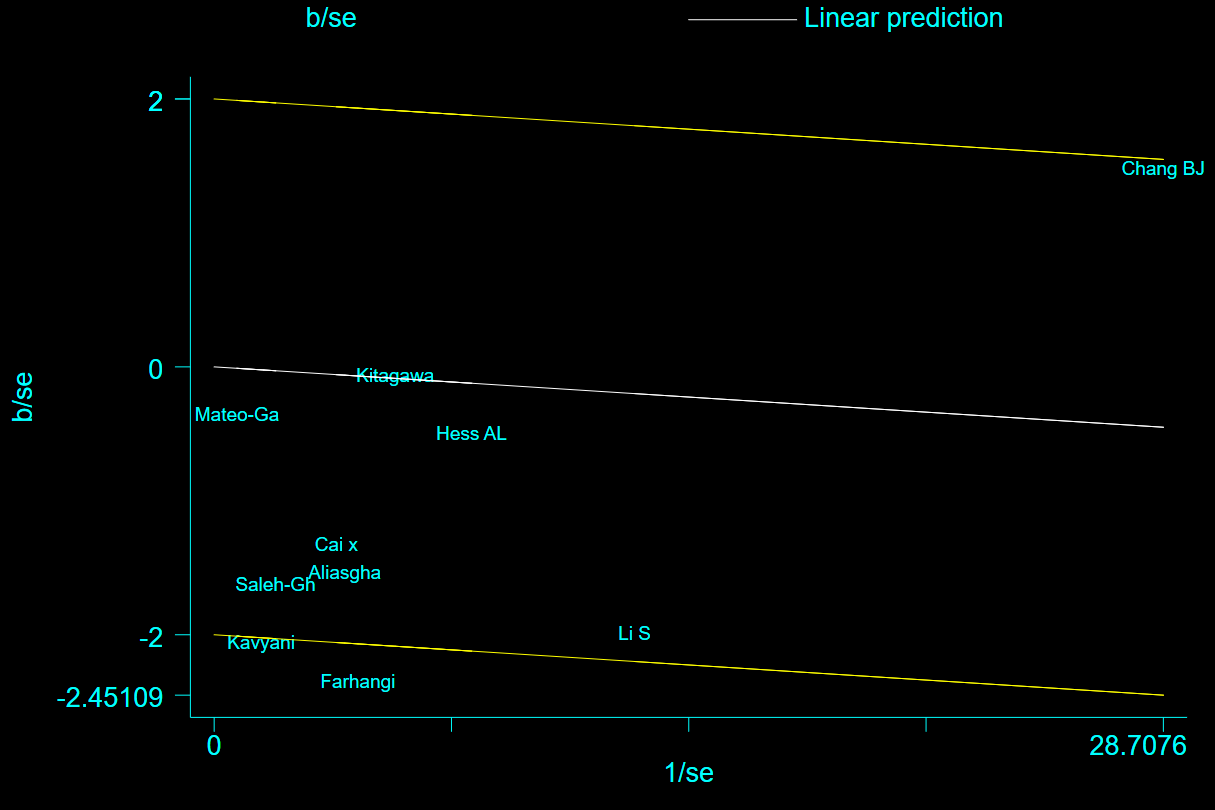

Supplement: Supplementary file 1 — Supplementary Material 1 [file 12937_2026_1292_MOESM1_ESM.zip › Supplementary Figure 7 HbA1c galb.tif]

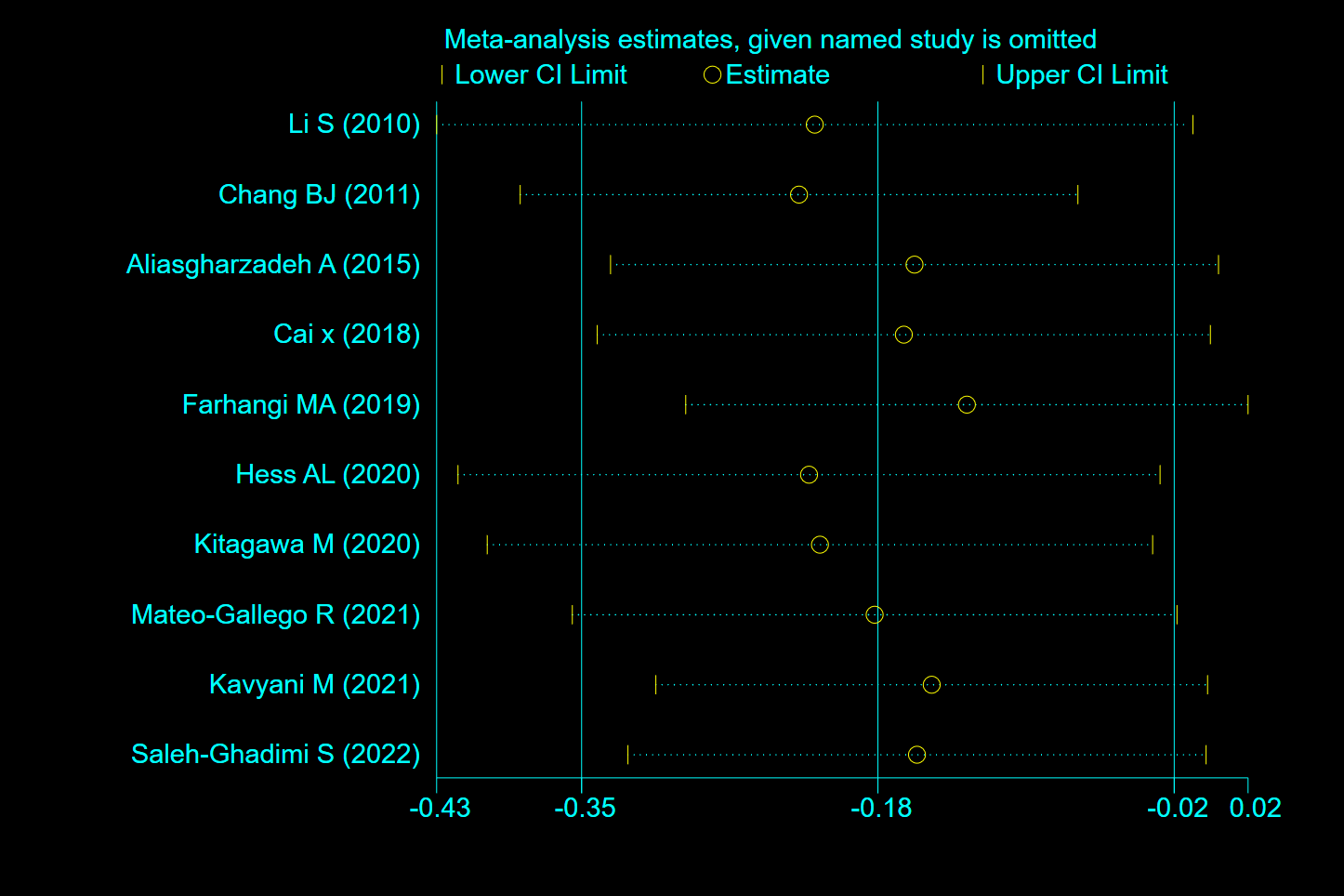

Supplement: Supplementary file 1 — Supplementary Material 1 [file 12937_2026_1292_MOESM1_ESM.zip › Supplementary Figure 8 HbA1c sensitive.tif]

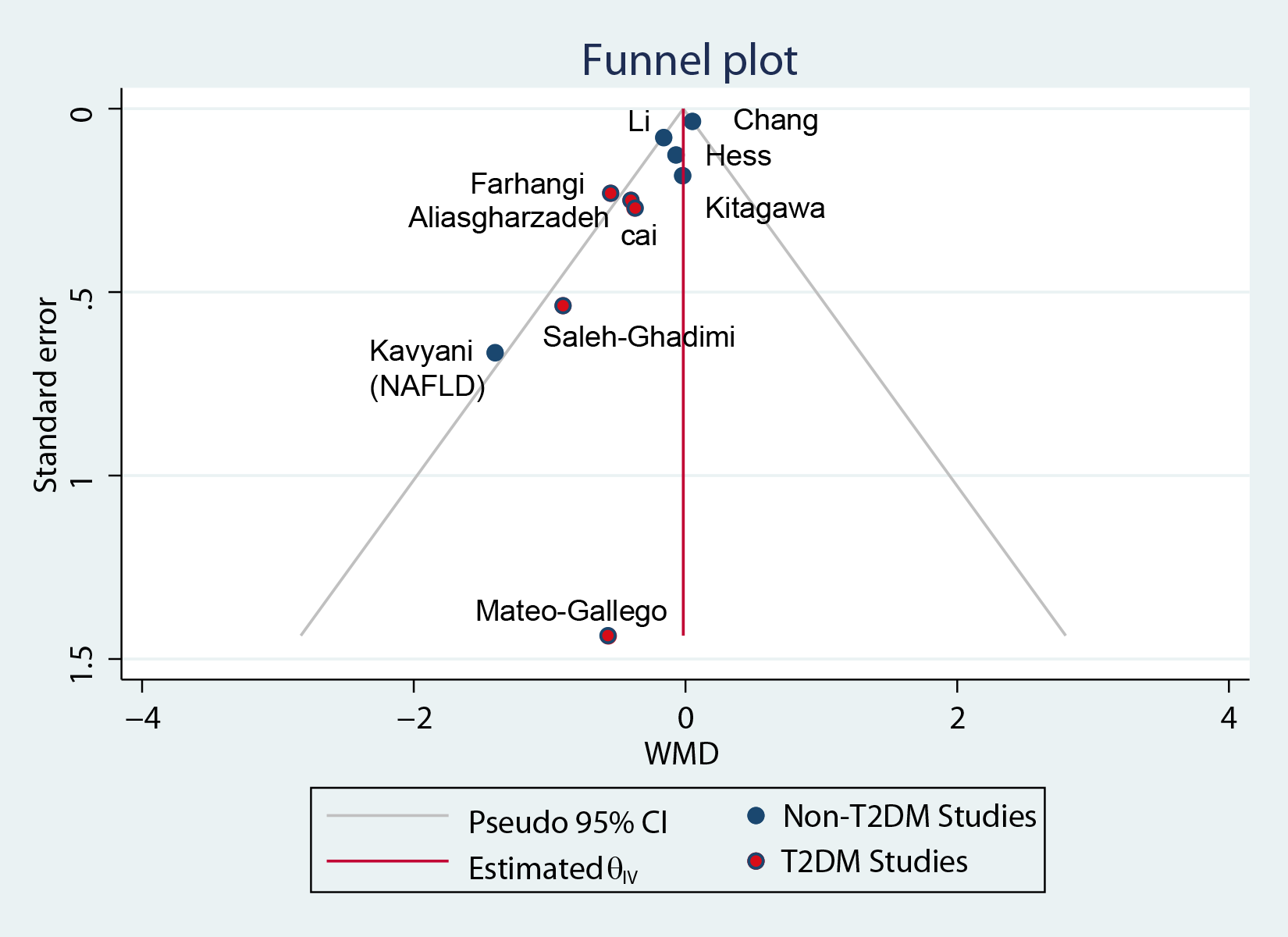

Supplement: Supplementary file 1 — Supplementary Material 1 [file 12937_2026_1292_MOESM1_ESM.zip › Supplementary Figure 9 HbA1c funnel.tif]
